# Confirming the presence of selected antibiotics and steroids in Norwegian biogas digestate

**DOI:** 10.1007/s11356-022-21479-1

**Published:** 2022-07-07

**Authors:** Astrid Solvåg Nesse, Stine Göransson Aanrud, Jan Ludvig Lyche, Trine Sogn, Roland Kallenborn

**Affiliations:** 1 Faculty of Environmental Sciences and Natural Resource Management, Norwegian University of Life Sciences, Ås, Norway; 2 Faculty of Veterinary Medicine, Norwegian University of Life Sciences, Ås, Norway; 3 Faculty of Chemistry, Biotechnology and Food Science, Norwegian University of Life Sciences, Ås, Norway

**Keywords:** Pharmaceuticals, Ecotoxicity, Risk assessment, Contamination, Environmental pollution

## Abstract

Farms utilizing sewage sludge and manure in their agronomic plant production are recognized as potential hotspots for environmental release of antibiotics and the resulting promotion of antibiotic resistance. As part of the circular economy, the use of biogas digestates for soil fertilizing is steadily increasing, but their potential contribution to the spreading of pharmaceutical residues is largely unknown. Digestates can be produced from a variety of biowaste resources, including sewage sludge, manure, food waste, and fish ensilage. We developed a method for the detection of 17 antibiotics and 2 steroid hormones and applied the method to detect pharmaceutical residues in digestates from most municipal biogas plants in Norway, covering a variety of feedstocks. The detection frequency and measured levels were overall low for most compounds, except a few incidents which cause concern. Specifically, relatively high levels of amoxicillin, penicillin G, ciprofloxacin, and prednisolone were detected in different digestates. Further, ipronidazole was detected in four digestates, although no commercial pharmaceutical products containing ipronidazole are currently registered in Norway. A simplified risk assessment showed a high risk for soil microorganisms and indicates the tendency for antibiotic-resistant bacteria for penicillin G and amoxicillin. For prednisolone and ipronidazole; however, no toxicity data is available for reliable risk assessments.

**Graphical abstract:**

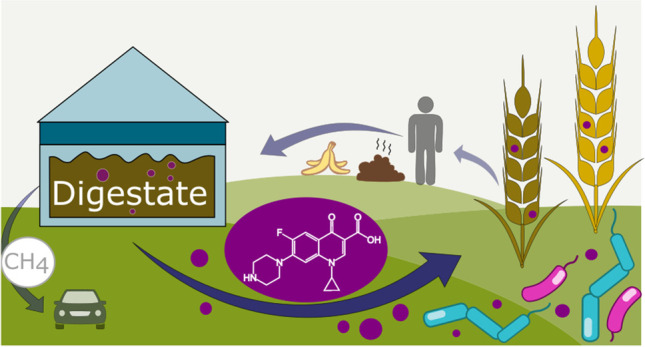

**Supplementary Information:**

The online version contains supplementary material available at 10.1007/s11356-022-21479-1.

## Introduction

Biogas production is increasingly used in circular bioeconomic strategies as a sustainable strategy for organic waste management. Currently, more than 18,000 biogas plants are registered in Europe (Cesaro, 2021). A variety of organic wastes such as sewage sludge and manure, as well as food and household wastes, are anaerobically digested for the production of biogas, containing mainly methane and carbon dioxide (the methane is thereafter used as an energy carrier). The nutrient-rich organic residue from that process is known as biogas digestate. While the use of biomethane for transport is well-known to reduce greenhouse gas emissions, Lyng et al. (2018) showed that the replacement of mineral fertilizer with biogas digestate is equally important for a reduced carbon footprint. Furthermore, the mineral phosphorus resources are finite with a proposed peak around 2030, and today, only 20% of all mined phosphorus is consumed in food (Childers et al. 2011). Thus, increased reuse of nutrients is important.

Unfortunately, in addition to the valuable nutrients, digestates have been shown to contain residues of legacy and emerging organic pollutants which are not fully degraded during the digestion process (Ali et al. 2019; Lindberg et al. 2005; Spielmeyer et al. 2014; Suominen et al. 2014; Widyasari-Metha et al. 2016). These anthropogenic contaminants may ultimately enter the agricultural production system if the digestates are spread on agricultural land (Chen et al. 2019).

Residues of antibiotics and steroids have been detected in sewage sludge, sewage effluents, manure, and other environmental matrices (Chang et al. 2007; Clarke and Smith 2011; Spielmeyer 2018; Verlicchi and Zambello 2015). Both antibiotics and steroid hormones, such as oestrogens and glucocorticoids, have well-known biological activity and may cause adverse environmental effects. For instance, continuous exposure of soil microbial communities to antibiotic agents by, e.g. manure, spread on agricultural land may lead to elevated levels of antibiotic resistance genes and resistant bacteria (Heuer et al. 2011). Further, the presence of antibiotics can disrupt the natural soil microbial flora and thereby adversely affect biogeochemical processes, such as nitrification, denitrification, and iron reduction (Grenni et al. 2018; Roose-Amsaleg and Laverman 2016). The exposure of aquatic organisms to glucocorticoids can lead to changes in behaviour and immunological responses, as demonstrated in previous studies (e.g. Bal et al. 2017; McNeil et al. 2016).

However, the fate of these contaminants in biogas digestate has only been sparsely investigated, due to lack of appropriate methods. Hence, we developed and validated a multi-compound quantitative trace level analytical method for the investigation of 16 antibiotics and steroid hormone residues in digestates. The choice of compounds was based on a former screening programme of the Norwegian Food Safety Authority. The list includes compounds considered important for food safety in Norway either due to frequent application in Norwegian agriculture or because they are banned for use in Norway according the EU regulation 37/2010/EC (NFSA 2015). The here developed method was applied to representative samples from 12 centralized municipal biogas plants (i.e. most of such plants in Norway) as well as two experimental reactors. Based on the detected pharmaceutical levels, a simplified risk assessment was performed by calculating expected soil concentration caused by the application of digestate and comparing those to ecotoxicity and antibiotic resistance development data.

## Materials and methods

### Collection of biogas digestates and information about the operating conditions

Biogas digestates were collected from twelve major municipal biogas plants in Norway (plants A to L, Tables 1 and S10), as well as two experimental units connected to research stations (plant M and plant I_exp_, connected to plant I). Each plant sampled about 1 L of their digestate and sent it to the Norwegian University of Life Sciences for quantitative analysis. Detailed information about the operating parameters, as supplied by the biogas plants, can be found in Table S10. The feedstocks used were food waste (E, G, and K), sewage sludge (D, H, J, and I), food waste mixed with sewage sludge (A, B, F, and L), manure mixed with food waste (C), manure (M), and a manure/fish silage combination (I_exp_). The digestates are referred to as liquid (< 5% dry matter, subscript L) or solid (20–50% dry matter, subscript S). Some plants produce both fractions; thus, the total number of digestates analysed was 18. Samples were quantified in duplicate for each digestate batch to account for method uncertainty and for the heterogeneity of the material. The same samples were previously analysed for pharmaceuticals and personal care products (PPCPs) by Ali et al. (2019).

**Table 1:** Overview of target compounds with their respective calibration curve range (ng mL^−1^ extract)

X-compounds	Y-compounds	Z-compounds
*Range: 0.25–30 ng mL*^*−1*^	*Range: 2.5–300 ng mL*^*−1*^	*Range: 10–600 ng mL*^*−1*^
*Sulfonamide antibiotics:*	*Fluoroquinolone antibiotics:*	*β-lactams antibiotics:*
Sulfadiazine	Ciprofloxacin	Amoxicillin
Sulfadoxine	Difloxacin	Penicillin G
Sulfamethazine	Enrofloxacin	
Sulfamethoxazole	Sarafloxacin	*Tetracycline antibiotics:*
	Norfloxacin	Chlortetracycline
*Nitroimidazole antibiotics:*		Doxycycline
Ipronidazole	*Glucocorticoids:*	Methacycline
Ipronidazole-OH	Dexamethasone	Oxytetracycline
Metronidazole	Hydrocortisone	Tetracycline
Ronidazole	Prednisolone	
2-Hydroxymethyl-1-methol-5-nitro-1H-imidazole		
(HMMNI)		
*Macrolide:*		
Tiamulin		
*Pyrimidine:*		
Trimethoprim		

### Target analytes

For the method validation process, 26 compounds from 8 classes of antibiotics and steroids were chosen as target analytes (Table 1). The target analytes have different linear concentration ranges as determined by the individual calibration curves, as well as different shelf life as standard solution. Based on the individual detector sensitivity and preservability, the compounds were divided into three groups: X, Y, and Z (Table 1). Separate solutions were prepared for each group, for both the native ^12^C standards (STDs) and the isotope-labelled internal standards (ISTDs). A complete list of target compounds including structure information and CAS registry numbers is available in Table S1. The stock solution concentrations and compositions are summarized in Table S2.

### Chemicals and solutions

Methanol (MeOH, HPLC-grade) and acetonitrile (ACN, HPLC-grade) were purchased from VWR (West Chester, PA, USA). Formic acid, ammonium acetate, disodium ethylene diamine tetra acetate (Na_2_EDTA), citric acid, sodium phosphate dibasic, and phosphoric acid were purchased from Sigma Aldrich (Oslo, Norway). Only grade 1 purified water from Milli-Q water purification systems (Millipore, Bedford, MA, USA) was used for the sample preparation and analysis. All standards and internal standards were purchased according to Table S1 in the Supplementary information. Separate solutions were prepared for the X-, Y-, and Z-compound groups both for the native ^12^C standards (STDs) and the isotope-labelled internal standards (ISTDs) (Table S2).

### Sample preparation and clean-up

**Fig. 1:**
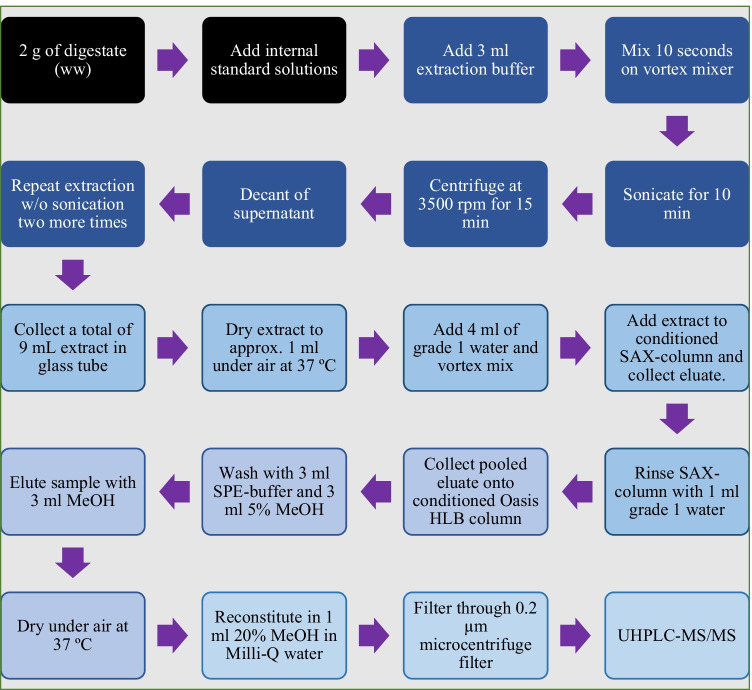
Flow chart for the extraction of antibiotics and corticoid steroids from biogas digestate, before quantitative analysis with UHPLC-MS/MS

Based on a previously published method (Hu et al. 2010), a comprehensive sample clean-up protocol was developed and validated ensuring minimum of matrix disturbances and co-elution in the final LC–MS/MS quantification (Fig. 1). An aliquot of 2 ± 0.03 g (wet weight, ww) digestate sample was weighed into 15 mL polypropylene tubes (Fig. 1). Internal standards were added, corresponding to 7.5 ng X-compound, 75 ng Y-compounds, and 150 ng Z-compounds (Table S2). Subsequently, 3 mL of extraction solution (Table S3) was added before the sample was vortexed for 10 s, ultrasonicated for 10 min, and centrifuged at 3500 rpm for 15 min. The supernatant was transferred to a glass tube, and the extraction procedure was repeated twice without ultrasonication. The combined supernatants were dried to approximately 1 mL with controlled heating at 37 °C under a stream of compressed air (analytical quality, AGA, Porsgrunn, Norway) using a Reacti-Therm III evaporator (Thermo Fisher Scientific Inc., Rockford, USA). The samples were subsequently shaken for 10 s with 4 mL of added grade 1 water. The extract, as well as 1 mL of water used to rinse the sample tube, was passed through a Bond Elut SAX column (500 mg, 3 mL) (Agilent, Santa Clara, USA) preconditioned with 2.5 mL MeOH and 2.5 mL grade 1 water, using light vacuum (water jet). The eluted extract was added to an Oasis HLB solid-phase extraction (SPE) column (200 mg, 6 mL) (Waters, Milford, USA) which was preconditioned with 5 mL MeOH and 5 mL water. The column was washed with 3 mL of SPE buffer (Table S3) and 3 mL of 5% MeOH in water. The sample was eluted with 3 mL MeOH and then evaporated to dryness at 37 °C. The sample was then reconstituted in 1 mL 20% MeOH in water and vortexed and filtered through a 0.2-μm microcentrifuge filter (Spin-X, Costar, Corning Inc. NY, USA) before the samples were transferred to 2-mL glass vials for quantitative analysis using LC–MS/MS. Triple quadrupole (QqQ) dynamic multiple reaction monitoring (dMRM) with electrospray ionization (MRM-ESI) was applied for quantitative analysis (Fig. 1).

### Analysis

Compound-specific chromatographic separation and quantitative detection of the cleaned digestate extracts were conducted on an Agilent 1260 ultra-high performance liquid chromatograph (UHPLC; Agilent Technologies, Waldbronn, Germany), and the detection and quantification were done on an Agilent 6490 triple quadrupole mass spectrometer (Agilent Technologies, Santa Clara, CA, USA) with an Agilent Jet Stream electrospray ion source, using dynamic multiple reaction monitoring (dMRM). For instrument control and method validation and quantification, the Agilent MassHunter software (V B.07.00/Build 7.0.457.0, 2008) was used. All MRM transitions and details from the MassHunter method, as well as the ion source parameters, are described in Tables S3 and S4 in the Supplementary information section.

### Method validation

For the method validation, detection limits, recovery, repeatability, accuracy, matrix effect, and efficiency of the extraction method were calculated. The details are given in the Supplementary information. Digestate are a complex matrix that, if not properly treated in advance, would affect the instrumental sensitivity and selectivity of each target analyte differently. To compensate for these variations, a matrix-matched calibration curve was chosen. The calibration curve was prepared with five concentration levels spanning the ranges given in Table 1, as well as one level with no added native compounds (level 0, see Table S5). Compounds were approved if the recovery rate was 40–115%, if the relative coefficient of variation (CV%) for the repeatability was < 15%, if the accuracy was <  ± 15% with a CV% < 15%, and if the determination coefficient (*R*^2^) of the matrix-matched calibration curve was above 0.985 (Table S6).

### Simplified risk assessment

For each of the quantified pharmaceuticals, the highest concentration found in any of the biogas digestates (C_DIG_) was used to calculate a predicted environmental concentration (PEC) in soil immediately after application of biogas digestate (Eq. 1). Application rates of digestate vary, due to, e.g. differences in nutrient and heavy metal content. Reasonable estimates according to the Norwegian Agricultural Extension Service is however 8 tonnes of solid or 45 tonnes of liquid digestates (fresh weight) per hectare (personal communication). Assuming an incorporation to a soil depth of 20 cm, and a dry soil bulk density of 1.3 kg l^−1^ (RHO_SOIL_), this corresponded to an application rate of 3.1 or 17.3 g fresh digestate kg^−1^ dry soil, respectively.

1 $$PEC\left[\mu g \cdot{kg}^{-1}\right]= {C}_{DIG} \left[ng \cdot{g}^{-1}\right]\times Application \, rate \left[g \cdot{kg}^{-1}\right]/ 1000 [ng\cdot {\mu g}^{-1}]$$

A predicted no-effect concentration (PNEC) for toxicity towards soil organisms was calculated following the standard approach of risk assessments from the European Chemicals Bureau (European Commission 2003): the lowest available EC50 or NOAEL value for soil organisms was divided by an assessment factor of 10–1000, depending on the availability of toxicity data. Tables S11 and S12 summarize the ecotoxicity data used for the PNEC calculations. For prednisolone, no soil toxicity data was available, and the PNEC_SOIL_ was derived by multiplying the PNEC_AQUATIC_ with the soil partitioning coefficient (*K*_d_); see Supplementary information for details. PNEC values for selection of antibiotic-resistant bacteria (ARB) were derived from Menz et al. (2019) by Eq. 2:

2 $${PNEC}_{ARB,SOIL}\left[\mu g \cdot{kg}^{-1}\right]=\frac{ {PNEC}_{ARB,PW}\left[\mu g\cdot {l}^{-1}\right] \times {K}_{d}\left[-\right]\times f(pw)(v/v)}{{RHO}_{SOIL}[kg\cdot {l}^{-1}]}$$

where *PNEC*_ARB,PW_ is the PNEC value in soil pore water, *K*_d_ is the soil–water distribution coefficient, and *f*(pw) is the fraction of pore water in the soil (0.25).

The risk quotient (RQ) was calculated as

3 $$RQ=PEC / PNEC$$

where a *RQ* < 0.1 indicates low risk, *RQ* = 0.1–1 indicates medium risk, and RQ > 1 indicates high risk.

## Results and discussion

### Method validation

Out of a total list of 26 selected target contaminants, 16 compounds were found to be valid for quantification (see Table 2). The detection and quantification thresholds for the validated compounds were highly to medium sensitive (in the pg g^−1^ to ng g^−1^ range), and their individual response curves were ranging over three orders of magnitude. The compounds are from six different compound classes, making this a multiclass quantification method appropriate for the screening in biogas digestate. Some of the compounds have lower recoveries than preferred, but since the quantification at ultra-trace level of these target chemicals (pg-range) is associated with an estimated overall method uncertainty between 40 and 50%, recovery rates between 40 and 115% were accepted. The results for all 26 compounds can be found in Table S6.

**Table 2:** Validation results of all successfully validated compounds, with recovery, method repeatability, method accuracy, matrix effect, and efficiency of the extraction method (EEM) at level 3 of the calibration curve (i.e. 7.5 ng mL^−1^ for X-compounds, 75 ng mL^−1^ for Y-compounds, and 150 ng mL^−1^ for the Z-compounds, see Table S5). *R*^2^ is the determination coefficient for the calibration curve

Compound	Recovery (mean ± SD)	Repeatability (CV%)	Accuracy (mean ± CV%)		*R*^2^	Matrix effect (%)	EEM (%)	Internal standard	Linear range	MDL	MQL
									ng g^−1^ digestate (fw)		
*Acceptable range*	40–115%	< 15%	± 15 ± 15%		> 0.985						
*Nitroimidazoles (X-type)*											
Ipronidazole	78 ± 3	3	− 2.8 ± 2	0.992		− 40	39	Ipronidazole-D_3_	0.4–15	0.13	0.40
Metronidazole	103 ± 3	3	4.2 ± 4	0.998		7	88	Metronidazole-^13^C_2_-^15^N_2_	0.4–15	0.13	0.38
Ronidazole	77 ± 5	5	− 5.0 ± 5	0.994		24	79	Ronidazole-D_3_	1.3–15	0.20	0.63
*Sulfonamides (X-type)*											
Sulfadiazine	96 ± 3	3	2.1 ± 3	0.997		87	55	Sulfadiazine-^13^C_6_	0.4–15	0.13	0.38
Sulfadoxine	74 ± 7	7	− 2.1 ± 7	0.993		− 12	21	Sulfadiazine-^13^C_6_	0.13–7.5	0.025	0.075
Sulfamethazine	111 ± 4	4	− 12 ± 4	0.995		61	62	Sulfadiazine-^13^C_6_	0.13–15	0.005	0.015
*Macrolide (X-type)*											
Tiamulin	81 ± 6	6	− 1.4 ± 6	0.994		− 56	32	Tiamulin-^13^C_4_	1.3–15	0.50	1.25
*Fluoroquinolones (Y-type)*											
Ciprofloxacin	43 ± 14	14	− 11 ± 13	0.990		− 22	9	Enrofloxacin-D_5_	3.8–150	1.3	3.8
Difloxacin	111 ± 3	3	8.2 ± 4	0.996		− 39	41	Difloxacin-D_3_	1.3–150	0.50	1.25
Enrofloxacin	101 ± 3	3	3.6 ± 3	0.995		− 33	23	Enrofloxacin-D_5_	1.3–150	0.20	0.63
Norfloxacin	113 ± 4	4	− 2.6 ± 4	0.993		3	12	Norfloxacin-D_5_	13–150	2.0	6.3
Sarafloxacin	68 ± 8	8	− 3.9 ± 8	0.994		− 23	14	Difloxacin-D_3_	1.3–150	1.3	3.8
*Corticosteroids (Y-type)*											
Prednisolone	95 ± 4	4	0.73 ± 4	0.991		− 39	192	Prednisolone-D_8_	13–150	2.0	6.3
Dexamethasone	93 ± 3	4	3.9 ± 3	0.995		− 36	188	Prednisolone-D_8_	1.3–150	0.25	0.75
*β-lactams (Y-type)*											
Amoxicillin	58 ± 4	4	5.8 ± 4	0.995		28	15	Amoxicillin-^13^C_6_	25–300	9.0	25
Penicillin G	43 ± 13	13	− 0.003 ± 13	0.991		− 32	16	Penicillin G-D_7_	5–300	1.5	5.0

When the matrix effect is ± 20%, no significant matrix effect is assumed. However, for most of the compounds, there was significant matrix effect confirmed, either signal enhancement (> 20%) or ion suppression (< − 20%). This demonstrates the need for a matrix-matched calibration curve. The efficiency of the extraction method was below 40% and above 115% for some compounds, demonstrating the need of well-defined internal standards. This also illustrates a considerable challenge for multi-compound quantification methods for trace level analysis. The deviating extraction efficiency of the compounds is all acceptable as long as the recovery is within the acceptable range, i.e. 40–115%.

### Levels of antibiotics and steroids in biogas digestate

Our survey revealed overall low levels of the target substances in the digestates. From the 16 target analytes, 8 were detected above the method quantification limit (MQL) (Table 3). Four were not detected in any of the samples, namely, ronidazole, enrofloxacin, sarafloxacin, and tiamulin. The antibiotics metronidazole, norfloxacin, difloxacin, and sulfadoxine and the glucocorticoid dexamethasone were detected in at least one biogas digestate, however, each below their respective MQLs. Despite overall low levels, a few findings were of concern. Amoxicillin, penicillin G, ciprofloxacin, and prednisolone were found at levels above 400 µg kg^−1^ dw. Also, ipronidazole was found in trace amounts in several digestates, even though the pharmaceutical is not registered for use in Norway.

**Table 3:** Concentration of selected antibiotics and steroid hormones in the digestates [μg kg^−1^ wet weight] (μg kg^−1^ dry weight is given in parenthesis, *n* = 2 except for E_L_ with *n* = 4). Operating parameters of the biogas plants A–N can be found in Table S10. Station codes equal those of Ali et al. (2019). Subscripts S and L denote solid and liquid digestates, respectively. The digestates are presented in the order of the substrate of their respective biogas plants. *3/4 replicates were < MQL. **The concentration of ciprofloxacin in digestate D_S_ was above the top concentration of the calibration curve, i.e. 150 μg kg.^−−1^ ww. *** Only one replicate was analysed. *AMX*, amoxicillin; *PENG*, penicillin G; *NOR*, norfloxacin; *CIP*, ciprofloxacin; *DFX*, difloxacin; *SDZ*, sulfadiazine; *SMZ*, sulfamethazine; *SDX*, sulfadoxine; *MET*, metronidazole; *IPRO*, ipronidazole; *PRED*, prednisolone; *DEXA*, dexamethasone. **Substrates: *L*, manure + food waste; *M*, manure; *N*, fish silage and manure (both substrate and digestate were analysed)

Sub	Station	AMX	PENG	NOR	CIP	DFX	SDZ	SMZ	SDX	MET	IPRO	PRED	DEXA
		*β-lactams*		*Fluoroquinolones*			*Sulfonamides*			*Nitroimidazoles*		*Glucocorticoids*	
Food waste	E_S_	121 ± 41(460)	< MDL	< MDL	< MDL	< MDL	< MDL	< MDL	< MDL	< MDL	< MDL	< MDL	< MQL
	E_L_	28 ± 9.7(960)*	< MDL	< MDL	< MDL	< MDL	< MDL	< MDL	< MDL	< MDL	< MDL	< MDL	< MQL
	G_L_	< MDL	< MDL	< MDL	< MDL	< MDL	< MDL	< MDL	< MDL	< MDL	< MDL	< MDL	< MQL
	K_S_	< MDL	< MDL	< MDL	< MDL	< MDL	< MDL	< MDL	< MDL	< MDL	< MDL	< MDL	< MQL
	K_L_	< MDL	< MDL	< MDL	< MDL	< MDL	3.6*** (140)	< MDL	< MDL	< MDL	< MDL	< MDL	< MQL
Sewage sludge	D_S_	< MDL	< MDL	< MQL	205 ± 22** (430)	< MQL	< MDL	0.039 ± 0.002 (0.08)	< MDL	< MDL	< MDL	< MDL	< MQL
	H_S_	< MDL	< MDL	< MDL	< MDL	< MDL	< MDL	< MDL	< MDL	< MDL	< MDL	< MDL	< MQL
	J_S_	< MDL	< MDL	< MQL	< MDL	< MDL	< MDL	< MDL	< MDL	< MDL	0.86 ± 0.16 (2.7)	< MDL	< MQL
	I_S_	< MDL	< MDL	< MDL	< MDL	< MDL	< MDL	< MDL	< MDL	< MDL	< MDL	< MDL	< MQL
Food waste + sewage sludge	F_S_	< MDL	< MDL	< MDL	< MDL	< MDL	< MDL	< MDL	< MDL	< MDL	< MDL	< MDL	< MQL
	F_L_	< MDL	< MDL	< MDL	< MDL	< MDL	< MDL	< MDL	< MDL	< MDL	< MDL	10.4 ± 1.0 (650)	< MQL
	A_S_	< MDL	< MDL	< MDL	< MDL	< MDL	< MDL	< MDL	< MDL	< MDL	0.77*** (1.6)	< MDL	< MQL
	A_L_	< MDL	< MDL	< MDL	< MDL	< MDL	< MDL	< MDL	< MDL	< MDL	< MQL	< MDL	< MQL
	B_S_	< MDL	< MDL	< MQL	< MDL	< MDL	< MDL	0.10 ± 0.002 (0.38)	< MDL	< MDL	< MDL	< MDL	< MDL
	L_S_	< MDL	< MDL	< MDL	< MDL	< MDL	< MDL	< MDL	< MQL	< MDL	< MQL	< MDL	< MDL
	L_L_	< MDL	< MDL	< MDL	< MDL	< MDL	< MDL	< MDL	< MDL	< MQL	< MDL	< MDL	< MQL
Others**	C_L_	< MDL	< MDL	< MDL	< MDL	< MDL	< MDL	0.079 ± 0.011 (1.6)	< MDL	< MDL	< MDL	< MDL	< MQL
	M_L_	< MQL	22 ± 7 (510)	< MDL	< MDL	< MDL	< MDL	< MDL	< MDL	< MDL	< MDL	< MDL	< MQL
	I_exp_ sub	< MDL	< MDL	< MDL	< MDL	< MDL	3.1 ± 0.08	0.40 ± 0.05	< MDL	< MDL	< MDL	< MDL	< MQL
	I_exp_ dig	< MDL	< MDL	< MDL	< MDL	< MDL	3.3 ± 0.24	0.43 ± 0.04	< MDL	< MDL	< MDL	< MDL	< MQL

#### β-lactams

Amoxicillin and penicillin G were found at 460–960 µg kg^−1^ dw in the food waste digestates E_S_ and E_L_ and the manure digestate M, respectively (Table 3). The detection of both antibiotics was surprising, as β-lactams are expected to rapidly degrade during biological processes by, e.g. hydrolysis (Braschi et al., 2013). The hydrolytic half-life of penicillin G is 60 h at 37 °C (Chadha et al., 2003), which is comparable to the temperature of reactor M (i.e. 35 °C). Consequently, β-lactams are rarely reported in manure despite their common use in animal husbandry. Hence, none of the recent reviews on antibiotics in manure by Spielmeyer (2018) and Wohde et al. (2016) reported the presence of either amoxicillin or penicillin G. Furthermore, the detection of amoxicillin in a digestate from food waste only was unexpected. Globally, antibiotics are frequently detected in animal products and vegetables, even as high as 1500–3000 μg kg^−1^ fw (in cultivated fish, China and Turkey, reviewed by Chen et al. (2019)), and He et al. found sulfonamides and fluoroquinolones in concentrations up to 15–20 μg l^−1^ in digested restaurant food waste in China. Such levels are, however, surprising for Norway where antibiotic consumption is generally reported as low, both in animal husbandry, fish farming, and human medicine (NIPH 2019; NORM/NORM-VET 2020). Usually, antibiotics are rarely detected above the maximum residue limits in Norwegian foodstuffs of animal origin (NFSA 2019).

Penicillin G is associated with a high risk quotient both towards soil bacteria (2.2, Table 4) and for development of antibiotic-resistant bacteria (ARB, 14.6), while the evaluation of amoxicillin resulted in lower risk quotients. The difference in risk quotients for ARB selection for amoxicillin and penicillin G is mainly due to the large difference in *K*_d_ values used in Eq. 2. These were estimated from their organic carbon partitioning constants (*K*_OC_) reported in a review by Cycoń et al. (2019); as for our study, no experimental *K*_d_ was available (details in Supplementary information). The *K*_OC_ used were 865.5 l kg^−1^ and 2.68 l kg^−1^ for amoxicillin and penicillin G, respectively. Consequently, penicillin G is predicted to sorb less to the soil and be as more bioavailable, as reflected in a lower PNEC value for penicillin G than for amoxicillin.

**Table 4:** Predicted environmental concentration (PEC), predicted no-effect concentration (PNEC) for soil organisms, and the corresponding risk quotients (RQ). *ARB*, antibiotic-resistant bacteria. PNEC values are based on Table S11, S12, and S15 in the Supplementary information

	PEC_SOIL_ [µg kg^−1^]	PNEC [µg kg^−1^] Soil organisms	RQ Soil organisms	PNEC [µg kg^−1^] ARB selection^2^	RQ ARB selection
Amoxicillin	0.660	0.47	1.4	6.7	0.099
Penicillin G	0.380	0.17	2.2	0.026	14.6
Sulfadiazine	0.062	5.6	0.01	31	0.002
Sulfamethazine	0.0012	10	0.0001		
Ciprofloxacin	0.462	0.5	0.9	32	0.014
Ipronidazole	0.003	NA	NA		
Prednisolone	0.180	0.09^1^	2^1^		

^1^Estimated from a simulated Koc value and from aquatic toxicity data using the partitioning coefficient method

^2^Derived from PNEC values in soil pore water estimated by Menz et al. (2019) (see Supplementary information)

#### Fluoroquinolones

Ciprofloxacin is one of the most examined and detected antibiotics worldwide (e.g. Verlicchi and Zambello 2015) and accounts for approximately 90% of the human consumption of quinolones in Norway (Sommerschild et al., 2020). Fluoroquinolones have a high affinity for sewage sludge (Lindberg et al. 2005), and ciprofloxacin was detected in all examined sewage sludges in Norway and Sweden in concentrations ranging from 70 to 770 μg kg^−1^ dw (TemaNord, 2012). Further, fluoroquinolones are persistent towards hydrolysis and high temperatures (Thiele-Bruhn 2003), and their degradation during anaerobic digestion is limited (Golet et al. 2003; Lindberg et al. 2006; Zhang and Li 2018). In fact, the concentration may even increase during thermal hydrolysis, probably due to release of intracellular antibiotics (Zhang and Li 2018). Ciprofloxacin can therefore be expected to be found in sewage sludge digestate, and the amount of 430 μg kg^−1^ dw found in digestate D_S_ in our study corresponds well with the range detected in sewage sludge by TemaNord (2012). A positive result was thus the lack of ciprofloxacin in 7 out of 8 biogas plants receiving sewage sludge, perhaps explained partly also by a decline in ciprofloxacin prescriptions of approximately 50% since 2012 as a strategy to prevent ciprofloxacin resistance (Sakshaug et al. 2017; Sommerschild et al. 2020).

In soils, fluoroquinolones are strongly sorbed to clay particles and organic matter, limiting their bioavailability and thus reducing their impact on soil biota and processes such as nitrogen transformation (Rosendahl et al., 2012). On the other side, the low availability combined with low hydrolysis and thermal and biological degradation (Al-Ahmad et al. 1999; Alexy et al. 2004; Thiele-Bruhn 2003) leads to their persistence in soil. Thus, they can accumulate when repeatedly added, as shown by Dalkmann et al. (2012), who found accumulation of ciprofloxacin in soils irrigated with wastewater. Further, Girardi et al. (2011) found that ciprofloxacin could inhibit soil respiration despite the formation of non-extractable residues. The risk quotient for ciprofloxacin was close to 1, indicating a moderate risk towards soil organisms.

It should be noted that ciprofloxacin was measured at a concentration more than twice as high as the upper boundary of the calibration curve. As the measured concentration of 430 µg kg^−1^ fw is uncertain, the upper boundary of 205 µg kg^−1^ fw was used for PEC_SOIL_ calculations. Thus, the risk quotient is probably underestimated. Considering its effect on soil organisms, persistence in soil, and the frequent detection in other studies, further investigations on the levels of ciprofloxacin in biogas digestates are needed.

#### Sulfonamides

Sulfonamides, represented by sulfadiazine and sulfamethazine, were found in several digestates. The concentrations were low, leading to low risk quotients. Neither sulfadiazine nor sulfamethazine was found in sewage sludge in the Nordic countries in 2012 (TemaNord 2012), probably because of their high hydrophobicity and low solid–liquid partition coefficients (*K*_d_) and because their negative charge at high pH hinders electrostatic sorption to the negatively charged surfaces of sludge (Göbel et al. 2005; Zhang and Li 2018). Sulfonamides are also used in veterinary medicine, but the detection frequency in German digestates from swine manure has been low (Spielmeyer et al. 2014).

#### Nitroimidazoles 

Ipronidazole was found at trace levels in four digestates from three different plants. Its presence is nevertheless surprising and concerning, as there are no registered pharmaceuticals in Norway for humans or animals containing ipronidazole (Østensen, H., personal communication, 16.07.2021). The digestates were based on sewage sludge alone (*J*_S_) or in combination with food waste (*A*_S_, *A*_L_, and *B*_S_) suggesting that human use is the origin of the pharmaceutical. For ipronidazole, there was no toxicological data available, and the risk quotient was not calculated.

#### Steroids

The glucocorticoid hormone prednisolone was found in one digestate sample only, while dexamethasone was found in trace amounts in almost all digestates. Presently, no toxicological information on prednisolone is reported for soil organisms, but studies on aquatic organisms have confirmed endocrine disrupting effects (Bal et al. 2017). Based on available toxicity data and *K*_d_ calculated from a modelled *K*_OC_ (see details in Supplementary information), a PNEC_SOIL_ of 0.09 μg kg^−1^ was calculated for prednisolone, yielding a risk quotient of 2. This number is only indicative but confirms the need of conducting in-depth toxicity tests for glucocorticoids and steroids on soil organisms as well as aquatic organisms. Dexamethasone has received increased attention as the World Health Organization is recommending dexamethasone to treat severe to critical COVID-19 cases (WHO 2020). This synthetic steroid is more effective compared to natural steroids but also more persistent in the environment due to the fluorine moiety in the molecule. As dexamethasone only was found in trace amounts, it was not done a risk evaluation of this compound.

## Concluding remarks

Food waste is an understudied matrix with regard to pharmaceutical pollution, as manure and sewage sludge are assumed to be more important entry routes to the soil. However, our results indicate that food waste can be an important entry route to the environment as well. Two other studies on pollutants in digestate from a variety of feedstocks (including food waste) also failed to identify a clear relationship between biogas feedstock and the level of pharmaceuticals and personal care products (PPCP) (Ali et al. 2019, using the same sample set as here) and POPs (Suominen et al. 2014).

As confirmed by our results, significant levels of pharmaceutical residues were detected in Norwegian biogas digestates despite well-established retainment technologies and national regulations for pharmaceuticals in veterinary and human medicine. Hence, it is likely that pharmaceutical residues are common in many biogas digestates in other countries as well. If untreated, the presence of antibiotics in biogas digestates may lead to increased antibiotic resistance, harm towards soil organisms, leaching to water bodies, and potential exposure of human consumers, when digestates are applied on agricultural land.

## Supplementary Information

Below is the link to the electronic supplementary material.

Detailed description of the method for analysis of antibiotics and hormones in biogas digestate, including quality control, operating conditions of the biogas plants, details of the target compounds, ecotoxicity data, Kd and Koc data, and calculations of PNECs. (DOCX 0.97 KB)

## Data Availability

All the research data used in the study are available in Table 1.

## References

[CR1] Al-Ahmad A., Daschner FD, Kümmerer K (1999) Biodegradability of cefotiam, ciprofloxacin, meropenem, penicillin G, and sulfamethoxazole and inhibition of waste water bacteria. Arch Environ Contam Toxicol 158–163 10.1007/s002449900501 10398765

[CR2] Alexy R, Kümpel T, Kümmerer K (2004). Assessment of degradation of 18 antibiotics in the Closed Bottle Test. Chemosphere.

[CR3] Ali AM, Nesse AS, Eich-Greatorex S, Sogn TA, Aanrud SG, Aasen Bunæs JA, Lyche JL, Kallenborn R (2019). Organic contaminants of emerging concern in Norwegian digestates from biogas production. Environ Sci Process Impacts.

[CR4] Bal N, Kumar A, Nugegoda D (2017). Assessing multigenerational effects of prednisolone to the freshwater snail, Physa acuta (Gastropoda: Physidae). J Hazard Mater.

[CR5] Braschi I, Blasioli S, Fellet C, Lorenzini R, Garelli A, Pori M, Giacomini D (2013). Persistence and degradation of new β-lactam antibiotics in the soil and water environment. Chemosphere.

[CR6] Cesaro A (2021). The valorization of the anaerobic digestate from the organic fractions of municipal solid waste : challenges and perspectives. J Environ Manage.

[CR7] Chadha R., Kashid N, Jain DVS (2003) Kinetic studies of the degradation of an aminopenicillin antibiotic (amoxicillin trihydrate) in aqueous solution using heat conduction microcalorimetry 1495–1503 10.1211/0022357022179 14713360

[CR8] Chang H, Hu J, Shao B (2007). Occurrence of natural and synthetic glucocorticoids in sewage treatment plants and receiving river waters. Environ Sci Technol.

[CR9] Chen J, Ying G, Deng W (2019). Antibiotic residues in food: extraction, analysis, and human health concerns. J Agric Food Chem.

[CR10] Childers DL, Corman J, Edwards M, Elser JJ (2011). Sustainability challenges of phosphorus and food: solutions from closing the human phosphorus cycle. Bioscience.

[CR11] Clarke BO, Smith SR (2011). Review of “emerging” organic contaminants in biosolids and assessment of international research priorities for the agricultural use of biosolids. Environ Int.

[CR12] Cycoń M, Mrozik A, Piotrowska-seget Z (2019). Antibiotics in the soil environment — degradation and their impact on microbial activity and diversity. Front Microbiol.

[CR13] Dalkmann P, Broszat M, Siebe C, Willaschek E, Sakinc T, Huebner J, Amelung W, Grohmann E, Siemens J (2012) Accumulation of pharmaceuticals, enterococcus, and resistance genes in soils irrigated with wastewater for zero to 100 years in central Mexico. PLoS One 7 10.1371/journal.pone.0045397 PMC3458031 23049795

[CR14] European Commission (2003) Technical guidance document on risk assessment in support of Commission Directive 93/67/EEC, Commission Regulation (EC) No 1488/94, and of Directive 98/8/EC. Part II. Luxembourg

[CR15] Girardi C, Greve J, Lamshöft M, Fetzer I, Miltner A, Schäffer A, Kästner M (2011). Biodegradation of ciprofloxacin in water and soil and its effects on the microbial communities. J Hazard Mater.

[CR16] Göbel A, Thomsen A, Mcardell CS, Joss A, Giger W (2005) Occurrence and sorption behavior of sulfonamides, macrolides, and trimethoprim in activated sludge treatment. Environ Sci Technol 3981–3989 10.1021/es048550a 15984773

[CR17] Golet EM, Xifra I, Siegrist H, Alder AC, Giger W (2003). Environmental exposure assessment of fluoroquinolone antibacterial agents from sewage to soil. Environ Sci Technol.

[CR18] Grenni P, Ancona V, Barra A (2018). Ecological effects of antibiotics on natural ecosystems : a review. Microchem J.

[CR19] Heuer H, Schmitt H, Smalla K (2011). Antibiotic resistance gene spread due to manure application on agricultural fields. Curr Opin Microbiol.

[CR20] Hu XY, Zhou Q, Luo Y (2010) Occurence and source analysis of typical veterinary antibiotics in manure, soil, vegetables and groundwater from organic vegetable bases, northern China. Environ Pollut 158:2992–2998 10.1016/j.envpol.2010.05.023 20580472

[CR21] Lindberg RH, Olofsson U, Rendahl P, Johansson MI, Tysklind M, Andersson BA (2006). Behavior of fluoroquinolones and trimethoprim during mechanical, chemical, and active sludge treatment of sewage water and digestion of sludge. Environ Sci Technol.

[CR22] Lindberg RH, Wennberg P, Johansson MI, Tysklind M, Andersson BAV (2005). Screening of human antibiotic substances and determination of weekly mass flows in five sewage treatment plants in Sweden. Environ Sci Technol.

[CR23] Lyng KA, Stensgård AE, Hanssen OJ, Modahl IS (2018). Relation between greenhouse gas emissions and economic profit for different configurations of biogas value chains: a case study on different levels of sector integration. J Clean Prod.

[CR24] McNeil PL, Nebot C, Sloman KA (2016). Physiological and behavioral effects of exposure to environmentally relevant concentrations of prednisolone during zebrafish (Danio rerio) embryogenesis. Environ Sci Technol.

[CR25] Menz J, Olsson O, Kümmerer K (2019) Antibiotic residues in livestock manure: does the EU risk assessment sufficiently protect against microbial toxicity and selection of resistant bacteria in the environment? J Hazard Mater 10.1016/j.jhazmat.2019.120807 31279308

[CR26] NFSA (2015) Overvåking av legemiddelrester og noen forurensende stoffer i animalsk mat og landdyr [Surveillance of pharmaceutical residues and some pollutants in animal food and land animals], the Norwegian Food Safety Authority

[CR27] NFSA (2019) The surveillance program of antibacterial residues in slaughtered cattle, sheep, goat and pig, the Norwegian Food Safety Authority

[CR28] NIPH (2019) 2019: use of pharmaceuticals in fish farming [WWW Document]. Nor. Inst. public Heal. URL https://www.fhi.no/hn/legemiddelbruk/fisk/2019-bruk-av-legemidler-i-fiskeoppdrett/ (accessed 11.27.20)

[CR29] NORM/NORM-VET (2020) Usage of antimicrobial agents and occurrence of antimicrobial resistance in Norway

[CR30] Roose-Amsaleg C, Laverman AM (2016). Do antibiotics have environmental side-effects? Impact of synthetic antibiotics on biogeochemical processes. Environ Sci Pollut Res.

[CR31] Rosendahl I, Siemens J, Kindler R, Groeneweg J, Zimmermann J, Czerwinski S, Lamshöft M, Laabs V, Wilke B, Vereecken H, Amelung W (2012). Persistence of the fluoroquinolone antibiotic difloxacin in soil and lacking effects on nitrogen turnover. J Environ Qual.

[CR32] Sakshaug S, Strøm H, Berg C, Blix HS, Litleskare I., Granum T (2017) Drug consumption in Norway 2012-2016. Oslo

[CR33] Sommerschild HT, Berg CL, Blix H, Litleskare I, Olsen K, Sharikabad MN, Amberger M, Torheim S, Granum T (2020) Drug consumption in Norway 2015-2019 - data from Norwegian drug wholesales statistics and the Norwegian prescription database. Oslo

[CR34] Spielmeyer A (2018). Occurrence and fate of antibiotics in manure during manure treatments: a short review. Sustain Chem Pharm.

[CR35] Spielmeyer A, Ahlborn J, Hamscher G (2014). Simultaneous determination of 14 sulfonamides and tetracyclines in biogas plants by liquid-liquid-extraction and liquid chromatography tandem mass spectrometry. Anal Bioanal Chem.

[CR36] Suominen K, Verta M, Marttinen S (2014). Hazardous organic compounds in biogas plant end products - soil burden and risk to food safety. Sci Total Environ.

[CR37] TemaNord (2012) PPCP monitoring in the Nordic countries-status report

[CR38] Thiele-Bruhn S (2003). Pharmaceutical antibiotic compounds in soils - a review. J Plant Nutr Soil Sci.

[CR39] Verlicchi P, Zambello E (2015). Pharmaceuticals and personal care products in untreated and treated sewage sludge : occurrence and environmental risk in the case of application on soil — a critical review. Sci Total Environ.

[CR40] WHO (2020) Corticosteroids for covid-19 [WWW Document]. World Heal. Organ. URL https://www.who.int/publications/i/item/WHO-2019-nCoV-Corticosteroids-2020.1

[CR41] Widyasari-Metha A, Hartung S, Kreuzig R (2016). From the application of antibiotic residues in liquid manueres and digestates: a screening study in one European center of conventional pig husbandry. J Environ Manage.

[CR42] Wohde M, Berkner S, Junker T, Konradi S, Schwarz L, Düring RA (2016) Occurrence and transformation of veterinary pharmaceuticals and biocides in manure: a literature review. Environ Sci Eur 28 10.1186/s12302-016-0091-8 PMC5044974 27761355

[CR43] Zhang X, Li R (2018). Variation of antibiotics in sludge pretreatment and anaerobic digestion processes: degradation and solid-liquid distribution. Bioresour Technol.

