# Supplementary material for: Confirming the presence of selected antibiotics and steroids in Norwegian biogas digestate
Source: Environ Sci Pollut Res Int. 2022 Jul 7;29(57):86595–605. doi: 10.1007/s11356-022-21479-1 (PMC9261245; doi:10.1007/s11356-022-21479-1)
Supplement: Supplementary file 1 — Detailed description of the method for analysis of antibiotics and hormones in biogas digestate, including quality control, operating conditions of the biogas plants, details of the target compounds, ecotoxicity data, Kd and Koc data, and calculations of PNECs. (DOCX 0.97 KB) [file 11356_2022_21479_MOESM1_ESM.docx]

**Supplementary information**

Confirming the presence of selected antibiotics and steroids in Norwegian biogas digestate

Astrid S. Nesse*^1^, Stine G. Aanrud^2^, Jan L. Lyche^2^, Trine A. Sogn^1^, Roland Kallenborn^3^

^1^Norwegian University of Life Sciences, Faculty of Environmental Sciences and Natural Resource Management
^2^Norwegian University of Life Sciences, Faculty of Veterinary Medicine
^3^Norwegian University of Life Sciences, Faculty of Chemistry, Biotechnology and Food Science
* email: [astrid.solvag.nesse@nmbu.no](mailto:astrid.solvag.nesse@nmbu.no)

**Overview of contents**

**S1** Target substances and internal standards
**S2** Solutions prepared for extraction
**S3** Ultra High-Performance Chromatograph /Triple Quadrupole mass spectrometry based quantification
**S4** Quality control
**S5** Validation results
**S6** Matrix effect of the validated analytes
**S7** Chromatography
**S8** Operating conditions at the biogas plants
**S90** Ecotoxicity tests and calculation of predicted no effect concentration (PNEC) values
**S10** Description of the performed statistics

**S1. Target substances and internal standards**

**Table S1.** Chemical structure and providing company, IUPAC name, and CAS-nr. for all tested compounds.

| **Compound (Abbreviation)** | **Molecular formula** | **Structure** | **CAS number** | **Description** | **Supplier** |
| --- | --- | --- | --- | --- | --- |
| HMMNI (HMMNI) | C_5_H_7_N_3_O_3_ | 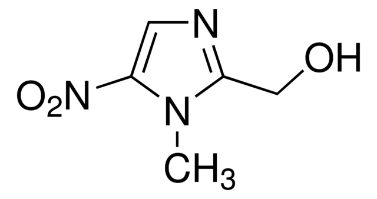 | 936-05-0 | Nitroimidazole, metabolite | Sigma Aldrich, Oslo, Norway |
| Ipronidazole (IPRO) | C_7_H_11_N_3_O_2_ | 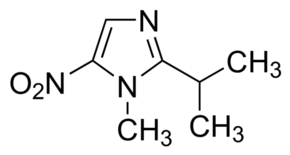 | 14885-29-1 | Nitroimidazole | Sigma Aldrich, Oslo, Norway |
| Ipronidazole-OH (IPOH) | C_7_H_11_N_3_O_3_ | 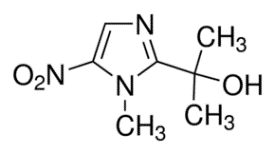 | 35175-14-5 | Nitroimidazole | Sigma Aldrich, Oslo, Norway |
| Metronidazole (MET) | C_6_H_9_N_3_O_3_ | 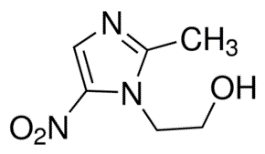 | 443-48-1 | Nitroimidazole | Sigma Aldrich, Oslo, Norway |
| Ronidazole (RDZ) | C_6_H_8_N_4_O_4_ | 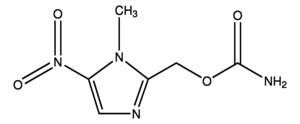 | 7681-76-7 | Nitroimidazole | Sigma Aldrich, Oslo, Norway |
| Sulfadiazine (SDZ) | C_10_H_10_N_4_O_2_S | 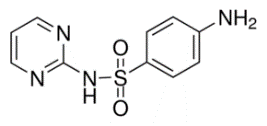 | 68-35-9 | Sulfonamide | Sigma Aldrich, Oslo, Norway |
| Sulfadoxine (SDX) | C_12_H_14_N_4_O_4_S | 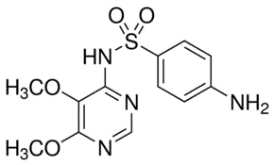 | 2447-57-6 | Sulfonamide | Sigma Aldrich, Oslo, Norway |
| Sulfamethazine (SMZ) | C_12_H_14_N_4_O_2_S | 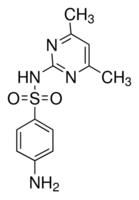 | 57-68-1 | Sulfonamide | Sigma Aldrich, Oslo, Norway |
| Sulfamethoxazole (SXZ) | C_10_H_11_N_3_O_3_S | 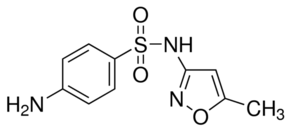 | 723-46-6 | Sulfonamide | Sigma Aldrich, Oslo, Norway |
| Tiamulin fumarate (TMN) | C_32_H_51_NO_8_S | 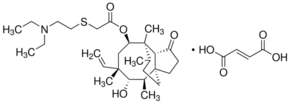 | 55297-96-6 | Macrolide | Sigma Aldrich, Oslo, Norway |
| Trimethoprim (TMP) | C_14_H_18_N_4_O_3_ | 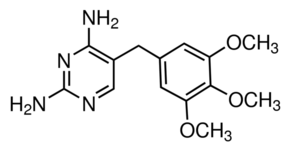 | 738-70-5 | Pyrimidine | Sigma Aldrich, Oslo, Norway |
| Ciprofloxacin (CIP) | C_17_H_18_FN_3_O_3_ | 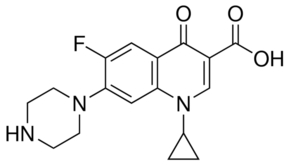 | 85721-33-1 | Fluoroquinolone | Sigma Aldrich, Oslo, Norway |
| Norfloxacin (NOR) | C_16_H_18_FN_3_O_3_ | 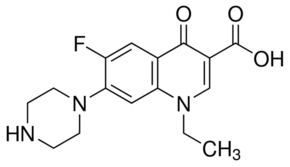 | 70458-96-7 | Fluoroquinoloe | Sigma Aldrich, Oslo, Norway |
| Difloxacin HCl (DFX) | C_21_H_19_F_2_N_3_O_3_ *HCl | 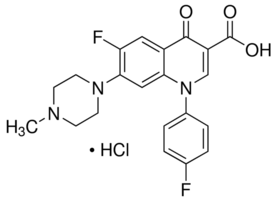 | 91296-86-5 | Fluoroquinolone | Sigma Aldrich, Oslo, Norway |
| Enrofloxacin (ENR) | C_19_H_22_FN_3_O_3_ | 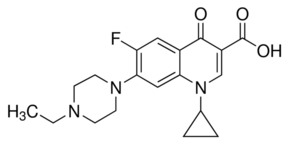 | 93106-60-6 | Fluoroquinolone | Sigma Aldrich, Oslo, Norway |
| Sarafloxacin HCL xH_2_O (SFX) | C_20_H_17_F_2_N_3_O_3_ *HCl *H_2_O | 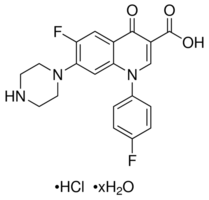 | 91296-87-6 (anhydrous) | Fluoroquinolone | Sigma Aldrich, Oslo, Norway |
| Dexamethazone (DEXA) | C_22_H_29_FO_5_ | 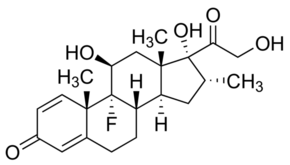 | 50-02-2 | Corticosteroide | Sigma Aldrich, Oslo, Norway |
| Hydrocortisone (HYCO) | C_21_H_30_O_5_ | 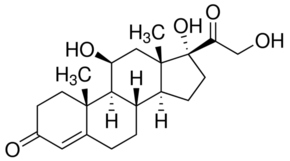 | 50-23-7 | Corticosteroide | Sigma Aldrich, Oslo, Norway |
| Prednisolone (PRED) | C_21_H_28_O_5_ | 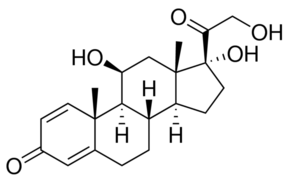 | 50-24-8 | Corticosteroide | Sigma Aldrich, Oslo, Norway |
| Amoxicillin trihydrat (AMX) | C_16_H_19_N_3_O_5_S *3H_2_O | 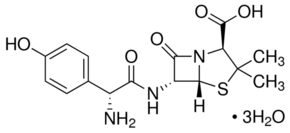 | [61336-70-7](https://www.sigmaaldrich.com/catalog/search?term=61336-70-7&interface=CAS%20No.&lang=en&region=US&focus=product) | β-lactam | Sigma Aldrich, Oslo Norway |
| Penicillin-G Potassium salt (PENG) | C_16_H_17_KN_2_O_4_S | 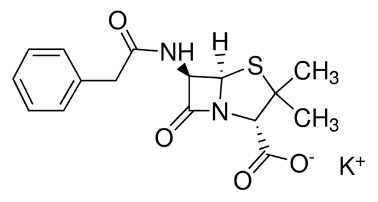 | 113-98-4 | β-lactam | Sigma Aldrich, Oslo, Norway |
| Chlortetracycline HCl (CTC) | C_22_H_23_ClN_2_O_8_ *HCl | 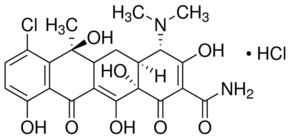 | 64-72-2 | Tetracycline | Sigma Aldrich, Oslo, Norway |
| Doxycyline hyclate (DC) | C_22_H_24_N_2_O_8_ *HCl * 0,5 H_2_O * 0,5 C_2_H_6_O | 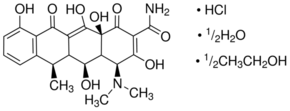 | 24390-14-5 | Tetracycline | Sigma Aldrich, Oslo, Norway |
| Methacycline HCl (MC) | C_22_H_22_N_2_O_8_ *HCl | 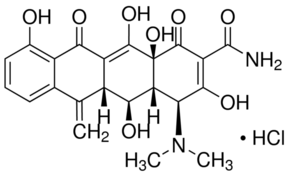 | 3963-95-9 | Tetracycline | Sigma Aldrich, Oslo, Norway |
| Oxytetracycline (OTC) | C_22_H_24_N_2_O_9_ | 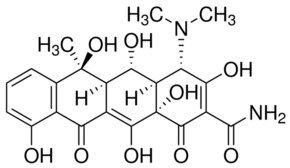 | 79-57-2 | Tetracycline | Sigma Aldrich, Oslo, Norway |
| Tetracycline HCl (TC) | C_22_H_24_N_2_O_8_ *HCl | 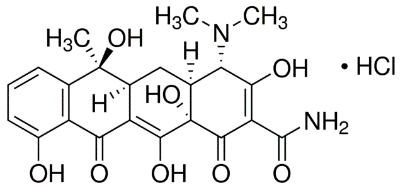 | 64-75-5 | Tetracycline | Sigma Aldrich, Oslo, Norway |

The internal standards HMMNI-D_3_, norfloxacin-D_5_, enrofloxacin-D_5_ HCl, difloxacin-D_3_ HCl 3xH_2_O, cortisol-D_4_, trimethoprim -D_9_, sulfadiazine phenyl-^13^C_6_, ronidazole-D_3,_ metronidazole ^13^C_2_-^15^N_2_, and ipronidazole-D_3_ were bought from Sigma Aldrich, Oslo, Norway. The internal standards tetracycline-D_6_ (80%), benzyl-penicillin-D_7_ potassium salt, amoxicillin ^13^C_6_, prednisolone-D_8_ (major), and tiamulin-^13^C_4_ fumarate were bought from Toronto Research Chemicals, Toronto Canada.

**S2. Solutions prepared for extraction**

Three solutions were prepared for the extraction of target compounds from the digestate. The ratios in the recipes are given on a volume basis. For an overview of X-, Y-, and Z-compounds, see Table S2. In addition, the ISTD stock solutions in Table S3 were prepared. The STD stock solutions in Table S3 were prepared for the calibration curves.

*Extraction solution* 1% formic acid in 50:50 ACN:MeOH
*McIlvaine buffer* 82% 0.2M Na_2_HPO_4_ and 18% 0.1M citric acid (pH 7)
*SPE-buffer* 1% 0.1M EDTA, 1% McIlvaine buffer, 2% MeOH, and 0.05% H_3_PO_4_ in grade 1 water.

**Table S2.** Stock solutions for the preparation of the calibration curves. STD stock A2 have ten times higher concentration of X- and Z-type compounds compared to STD stock A1, to cover the concentration span of the calibration curve.

| **STD stock A1 and STD stock A2** | | | **STD stock B** |
| --- | --- | --- | --- |
| *A1: 10 ng mL^-1^ / A2: 100 ng mL^-1^* | | *A1: 100 ng mL^-1^ / A2: 1000 ng mL^-1^* | *1000 ng mL^-1^* |
| HMMNI | | Ciprofloxacin | Amoxicillin |
| Ipronidazole | | Sarafloxacin | Penicillin G |
| Ipronidazole-OH | | Difloxacin | Doxycycline |
| Metronidazole | | Enrofloxacin | Methacycline |
| Ronidazole | | Norfloxacin | Oxytetracycline |
| Sulfadiazine | | Dexamethasone | Chlortetracycline |
| Sulfadoxine | | Prednisolone | Tetracycline |
| Sulfamethazine | | Hydrocortisone |  |
| Sulfamethoxazole | |  |  |
| Tiamulin | |  |  |
| Trimethoprim | |  |  |
| **ISTD stock A** | | | **ISTD stock B** |
| *100 ng mL^-1^* | *1 µg mL^-1^* | | *1 µg mL^-1^* |
| HMMNI-D_3_ | Cortisol-D_4_ | | Amoxicillin-^13^C_6_ |
| Ipronidazole-D_3_ | Difloxacin-D_3_ | | Penicillin G-D_7_ |
| Metronidazole-^13^C_2_-^15^N_2_ | Enrofloxacin-D_5_ | | Tetracycline-D_6_ |
| Ronidazole-D_3_ | Norfloxacin-D_5_ | |  |
| Sulfadiazine-^13^C_6_ | Prednisolone-D_8_ | |  |
| Tiamulin-^13^C_4_ |  | |  |
| Trimethoprim-D_9_ |  |  |  |

**S3. Ultra High-Performance Liquid Chromatograph / Triple Quadrupole mass spectrometry based quantification**

**Table S3.** Ion source parameters

| **Parameter** | **Value** | **Unit** |
| --- | --- | --- |
| Gas Temp: | 180 | °C |
| Gas Flow | 19 | l/min |
| Nebulizer | 35 | psi |
| Sheath Gas Temp | 350 | °C |
| Sheath Gas flow | 12 | l/min |
| Capillary | 3000 | V |
| Nozzle Voltage | 0 | V |
| High Pressure RF | 150 | V |
| Low Pressure RF | 70 | V |

**Table S4.** Chosen MS-parameters and multiple reaction monitoring transitions (MRMs) tested for validation, with compound type. Retention time (RT) and delta retention time (ΔRT) are given in minutes. Validated quantifier transitions are marked bold with grey background. Validated qualifier transitions are marked in bold. For internal standards, the monitoring transition validated for the analytes’ quantifier transition are marked with grey background. Collision energy (CE) are given in Volt. Cell Accelerator voltage (CAV) is the voltage gradient in the collision cell.

| **Type** | **Compound Name** | **RT (min)** | **ΔRT** | **Precursor Ion** | **Product Ion** | **CE** | **CAV** |
| --- | --- | --- | --- | --- | --- | --- | --- |
| X | HMMNI | 1.9 | 1 | 158.0 | 139.8 | 10 | 2 |
|  |  |  |  | 158.0 | 55.2 | 20 | 3 |
| X | HMMNI-D_3_ (ISTD) | 1.9 | 1 | 161.0 | 143.0 | 10 | 2 |
|  |  |  |  | 161.0 | 58.0 | 19 | 2 |
| X | Ipronidazole | 6.2 | 1 | **170.0** | **108.9** | 26 | 5 |
|  |  |  |  | **170.0** | **124.1** | 20 | 3 |
| X | Ipronidazole-D_3_ (ISTD) | 6.2 | 1 | 173.0 | 112.0 | 29 | 2 |
|  |  |  |  | 173.0 | 127.0 | 18 | 2 |
| X | Ipronidazole-OH | 5.0 | 1 | 186.0 | 121.0 | 30 | 3 |
|  |  |  |  | 186.0 | 168.0 | 10 | 7 |
| X | Metronidazole | 2.1 | 1 | **172.0** | **82.0** | 26 | 7 |
|  |  |  |  | **172.0** | **128.0** | 12 | 4 |
| X | Metronidazole-^13^C_2_-^15^N_2_ (ISTD) | 2.1 | 1 | 176.0 | 86.0 | 27 | 3 |
|  |  |  |  | 176.0 | 132.0 | 13 | 3 |
| X | Ronidazole | 2.1 | 1 | **201.0** | **55.1** | 30 | 3 |
|  |  |  |  | **201.0** | **139.9** | 10 | 2 |
| X | Ronidazole-D_3_  (ISTD) | 2.1 | 1 | 204.0 | 58.0 | 21 | 2 |
|  |  |  |  | 204.0 | 113.0 | 15 | 2 |
|  |  |  |  | 204.0 | 143.0 | 7 | 2 |
| X | Sulfadiazine | 2.0 | 1 | **251.0** | **91.8** | 23 | 7 |
|  |  |  |  | 251.0 | 107.6 | 22 | 5 |
|  |  |  |  | **251.0** | **156.0** | 11 | 2 |
| X | Sulfadiazine-^13^C_6_  (ISTD) | 2.0 | 1 | 257.0 | 98.0 | 27 | 2 |
|  |  |  |  | 257.0 | 114.0 | 25 | 2 |
|  |  |  |  | 257.0 | 162.0 | 14 | 3 |
| X | Sulfadoxine | 5.5 | 1 | **311.0** | **107.7** | 26 | 5 |
|  |  |  |  | **311.0** | **156.0** | 10 | 8 |
| X | Sulfamethazine | 3.9 | 1 | 279.1 | 91.9 | 32 | 6 |
|  |  |  |  | **279.1** | **123.9** | 23 | 3 |
|  |  |  |  | **279.1** | **185.9** | 15 | 6 |
| X | Sulfamethoxazole | 5.1 | 1 | 254.0 | 92.0 | 27 | 4 |
|  |  |  |  | 254.0 | 108.0 | 22 | 4 |
|  |  |  |  | 254.0 | 156.0 | 11 | 2 |
| X | Tiamulin | 9.0 | 1 | **494.0** | **118.8** | 41 | 4 |
|  |  |  |  | **494.0** | **192.0** | 20 | 5 |
| X | Tiamulin-^13^C_4_ (ISTD) | 9.0 | 1 | 498.0 | 119.0 | 45 | 4 |
|  |  |  |  | 498.0 | 196.0 | 20 | 4 |
| X | Trimethoprim | 3.5 | 1 | 291.1 | 122.9 | 35 | 4 |
|  |  |  |  | 291.1 | 230.0 | 21 | 3 |
|  |  |  |  | 291.1 | 260.9 | 23 | 2 |
| X | Trimethoprim-D_9_ (ISTD) | 3.5 | 1 | 300.0 | 123.0 | 24 | 2 |
|  |  |  |  | 300.0 | 234.0 | 25 | 2 |
|  |  |  |  | 300.0 | 264.0 | 28 | 2 |
| Y | Ciprofloxacin | 5.0 | 1.5 | **332.1** | **230.9** | 38 | 4 |
|  |  |  |  | **332.1** | **287.9** | 16 | 7 |
|  |  |  |  | 332.1 | 314.0 | 19 | 6 |
| Y | Cortisol-D_4_  (ISTD) | 9.4 | 1 | 367 | 121.0 | 26 | 4 |
|  |  |  |  | 367 | 327.2 | 16 | 2 |
|  |  |  |  | 367 | 349.0 | 14 | 2 |
| Y | Dexamethasone | 9.7 | 1 | **393.1** | **146.9** | 33 | 8 |
|  |  |  |  | **393.1** | **373.0** | 3 | 4 |
| Y | Difloxacin | 5.6 | 1 | **400.0** | **298.9** | 28 | 7 |
|  |  |  |  | **400.0** | **355.9** | 18 | 5 |
|  |  |  |  | 400.0 | 381.9 | 28 | 4 |
| Y | Difloxacin-D_3_ (ISTD) | 5.6 | 1 | 403.0 | 299.0 | 34 | 2 |
|  |  |  |  | 403.0 | 359.0 | 20 | 2 |
|  |  |  |  | 403.0 | 385.0 | 23 | 4 |
| Y | Enrofloxacin | 5.2 | 1 | **360.0** | **245.0** | 25 | 3 |
|  |  |  |  | 360.0 | 315.8 | 18 | 6 |
|  |  |  |  | **360.0** | **341.9** | 19 | 5 |
| Y | Enrofloxacin-D_5_ (ISTD) | 5.2 | 1 | 365.0 | 245.0 | 30 | 2 |
|  |  |  |  | 365.0 | 321.0 | 21 | 2 |
|  |  |  |  | 365.0 | 347.0 | 23 | 3 |
| Y | Hydrocortisone | 9.4 | 1 | 363.0 | 97.0 | 26 | 5 |
|  |  |  |  | 363.0 | 121.0 | 20 | 3 |
|  |  |  |  | 363.0 | 327.2 | 13 | 5 |
| Y | Norfloxacin | 4.8 | 1.5 | **320.0** | **189.0** | 53 | 6 |
|  |  |  |  | **320.0** | **230.7** | 48 | 3 |
|  |  |  |  | 320.0 | 301.9 | 25 | 6 |
| Y | Norfloxacin-D_5_ (ISTD) | 4.8 | 1.5 | 325.0 | 238.0 | 28 | 2 |
|  |  |  |  | 325.0 | 281.0 | 18 | 2 |
|  |  |  |  | 325.0 | 307.0 | 20 | 3 |
| Y | Prednisolone | 9.3 | 1 | **361.0** | **147.0** | 30 | 2 |
|  |  |  |  | 361.0 | 325.0 | 10 | 2 |
|  |  |  |  | **361.0** | **343.0** | 5 | 3 |
| Y | Prednisolone-D_8_ (ISTD) | 9.3 | 1 | 369.0 | 151.0 | 35 | 3 |
|  |  |  |  | 369.0 | 332.0 | 8 | 3 |
|  |  |  |  | 369.0 | 351.0 | 7 | 2 |
| Y | Sarafloxacin | 6.0 | 1 | **386.0** | **342.0** | 17 | 5 |
|  |  |  |  | **386.0** | **367.7** | 20 | 4 |
| Z | Amoxicillin | 1.6 | 1 | 366.0 | 113.9 | 22 | 3 |
|  |  |  |  | **366.0** | **134.0** | 29 | 3 |
|  |  |  |  | 366.0 | 208.0 | 8 | 4 |
| Z | Amoxicillin-^13^C_6_ (ISTD) | 1.6 | 1 | 372.0 | 114.0 | 20 | 4 |
|  |  |  |  | 372.0 | 213.0 | 16 | 2 |
|  |  |  |  | 372.0 | 355.0 | 6 | 2 |
| Z | Chlortetracycline | 7.2 | 2 | 479.0 | 154.0 | 30 | 2 |
|  |  |  |  | 479.0 | 444.0 | 20 | 2 |
|  |  |  |  | 479.0 | 462.0 | 15 | 2 |
| Z | Doxycycline | 8.4 | 2 | 445.0 | 98.0 | 46 | 6 |
|  |  |  |  | 445.0 | 321.0 | 33 | 6 |
|  |  |  |  | 445.0 | 428.0 | 17 | 2 |
| Z | Methacycline | 8.1 | 2 | 443.0 | 200.7 | 32 | 6 |
|  |  |  |  | 443.0 | 425.8 | 16 | 3 |
| Z | Oxytetracycline | 4.8 | 2 | 461.0 | 200.6 | 33 | 6 |
|  |  |  |  | 461.0 | 425.8 | 18 | 3 |
|  |  |  |  | 461.0 | 443.3 | 12 | 2 |
| Z | Pencillin G | 9.0 | 1 | **335.0** | **114.0** | 27 | 6 |
|  |  |  |  | **335.0** | **160.0** | 10 | 7 |
|  |  |  |  | 335.0 | 176.0 | 12 | 8 |
| Z | Penicillin G-D_7_ (ISTD) | 9.0 | 1 | 342.0 | 114.0 | 35 | 3 |
|  |  |  |  | 342.0 | 160.0 | 10 | 3 |
|  |  |  |  | 342.0 | 183.0 | 11 | 2 |
| Z | Tetracycline | 4.6 | 2 | 445.0 | 153.9 | 28 | 2 |
|  |  |  |  | 445.0 | 409.9 | 17 | 3 |
|  |  |  |  | 445.0 | 427.0 | 10 | 5 |
| Z | Tetracycline-D_6_ (ISTD) | 4.6 | 2 | 451.0 | 160.0 | 30 | 4 |
|  |  |  |  | 451.0 | 416.0 | 18 | 3 |
|  |  |  |  | 451.0 | 433.0 | 12 | 2 |

**S4. Quality control**

***Detection limits***

The method detection limit (MDL) and method quantification limit (MQL) were determined by preparing and injecting three spiked blank matrix samples with varying concentration below the lowest concentration in the calibration curve (i.e., below level 1, Table 3), measuring signal and noise, and determining when signal to noise (S/N) was above 3 for MDL or above 10 for MQL.

***Matrix matched calibration curve***

) The concentration levels of the matrix matched calibration curve are found in Table S6. To make the matrix matched calibration curve, STDs and ISTDs were added to digestate samples prior to the sample preparation procedure (Figure S1), with two replicates of each level. Four additional replicates were made at level 3, to have six replicates for the calculations of recovery, repeatability, accuracy, and efficiency of extraction method.

In addition, a blank matrix sample (n=1) and a method blank (n=1) was prepared. The blank matrix sample was prepared by extracting digestate with no added native standards (STDs) or internal standards (ISTDs). The purpose was to examine whether any matrix components would give artificial peaks, especially for the ISTDs. Method blank was prepared by adding ISTDs to 2 mL grade 1 water prior to sample preparation. The method blank was used to control whether the sample preparation procedure would produce any peak for the analytes.

**Table S5.** Concentration levels of target compounds (STD) and isotope labelled internal standards (ISTD) in the matrix matched calibration curve. The ISTD were added in equal concentration to all six levels.

|  |  | **STD (ng mL^-1^)** | | | | | | **ISTD (ng mL^-1^)** |
| --- | --- | --- | --- | --- | --- | --- | --- | --- |
| **Level** | | **0** | **1** | **2** | **3** | **4** | **5** |  |
| X-compounds | | 0 | 0.25 | 2.5 | 7.5 | 15 | 30 | 7.5 |
| Y-compounds | | 0 | 2.5 | 25 | 75 | 150 | 300 | 75 |
|  | |  |  |  |  |  |  |  |
| Z-compounds | | 0 | 10 | 50 | 150 | 300 | 600 | 150 |

As the calibration curve spans over several order of magnitudes in concentration, five solutions of STDs and ISTDs containing X+Y or Z compounds (Table S2) in different concentrations were used to prepare the matrix matched calibration curve. The Z-compounds are kept in separate solutions, as they need to be prepared fresh before every analysis day due to their short shelf life.

***Recovery, repeatability, and accuracy***

Recovery was calculated according to equation 1, where *signal (I)STD* refers to the actual signal level and *conc* refers to the expected signal level (i.e., level 3, Table S6). *Matrix* refers to the six replicates at level 3 in the matrix matched calibration curve, *blank* refers to the blank matrix sample (n = 1). The *solvent* sample (n = 1) was prepared by adding STDs corresponding to level 3 (Table S6) to 20% MeOH. The standard deviation for the recovery are reported as well (Table S7).

$Recovery \left( \% \right)=\sum_{i=1}^{n=6} \frac{\left( \frac{{signal STD \left( matrix \right)}_{i}}{{signal ISTD\left( matrix \right)}_{i}} - \frac{signal \left( blank \right)}{signal ISTD\left( blank \right)} \right) * conc\left( solvent \right)}{\frac{signal STD \left( solvent \right)}{signal ISTD\left( solvent \right)} * conc\left( sample \right)}*\frac{100}{n}$ (eq. 1)

Repeatability is reported as the relative coefficient of variation (CV%) of the six matrix samples at level 3 according to equation 2 and 3, where $x$ is the signal of the six matrix matched samples at level 3 and N is six.

$\sigma= \sqrt{\frac{1}{N-1} \sum_{i=1}^{N} {(x_{i}- \bar{x})}^{2}}$ (eq. 2)

$CV\%=\frac{\sigma}{\bar{x}}*100\%$ (eq. 3)

Accuracy was calculated as the average difference between calculated concentration and expected concentration for each of the six level 3 samples and are reported in % of expected concentration (equation 4). The relative coefficient of variation (CV%) was calculated and are reported in the results as well (Table S7).

$Accuracy \left( \% \right)= \sum_{i=1}^{n=6} {signal STD(matrix)}_{i}-conc\left( sample \right)*100\% / n$ (eq. 4)

The calculations for recovery, repeatability, and accuracy were done for each monitored transition for all target analytes.

**S5. Result validation**

The initially chose target analytes were validated if they passed four criteria: (1) Recovery rate in the range 40 to 115%, (2) The relative coefficient of variation (CV%) for the repeatability was below 15%, (3) The accuracy was below 15% with a CV < 15%, and (4) The determination coefficient (R^2^) of the matrix matched calibration curve was above 0.985.

Several target substances did not meet the four quality criteria thresholds. All compounds marked in grey (Table S7) were rejected for further validation. This includes HMMNI (1-methyl-5-nitro-1H-imidazol-2-yl-methanol), for which no validated qualifier MRM-transition was found in the digestate, as well as hydrocortisone, which had a significant matrix-associated contamination in combination with low recovery rates during the matrix spiking experiment.

The quantification frame defined by linearity range, method detection limit (MDL), and method quantification limit (MQL) for the individual analytes is summarized in Table 5, where an overview over all approved compounds are given. In general, highly sensitive thresholds were achieved (pg g^-1^ to ng g^-1^ range) and individual linear response ranges over three orders of magnitudes are reached.

**Table S6.** Validation results with recovery, method repeatability, and method accuracy at level 3 (7.5 ng mL^-1^ for X-compounds, 75 ng mL^-1^ for Y-compounds and 150 ng mL^-1^ for Z-compounds, see Table 3), as well as the determination coefficient (R^2^) for the calibration curve. Targets marked with grey did not meet the quality control criteria. *Tetracycline, with an accepted linear range from 150 – 300 ng/mL was not approved as validated.

| **Type** | **Compound** | **Recovery (**mean ± sd) | **Repeatability** (CV%) | **Accuracy** (mean ± CV%) | **R^2^** |
| --- | --- | --- | --- | --- | --- |
| ***Acceptable range*** | | **40-115%** | **<15%** | **±15 ±15%** | **>0.985** |
| X | 2-Hydroxymethyl-1-methyl-5-nitro-1*H*-imidazole (HMMNI) | 135±15 | 18 | 7.1±1 | 0.994 |
| X | Ipronidazole | 78±3 | 3 | -2.8±2 | 0.992 |
| X | Ipronidazole-OH | 214±7 | 7 | 4.7±7 | 0.996 |
| X | Metronidazole | 103±3 | 3 | 4.2±4 | 0.998 |
| X | Ronidazole | 77±5 | 5 | -5.0±5 | 0.994 |
| X | Sulfadiazine | 96±3 | 3 | 2.1±3 | 0.997 |
| X | Sulfadoxine | 74±7 | 7 | -2.1±7 | 0.993 |
| X | Sulfamethazine | 111±4 | 4 | -12±4 | 0.995 |
| X | Sulfamethoxazole | 40±10 | 10 | 38±14 | 0.924 |
| X | Tiamulin | 81±6 | 6 | -1.4±6 | 0.994 |
| X | Trimethoprim | 105±17 | 17 | 27±22 | 0.950 |
| Y | Ciprofloxacin | 43±14 | 14 | -11±13 | 0.990 |
| Y | Dexamethasone | 93±3 | 4 | 3.9±3 | 0.995 |
| Y | Difloxacin | 111±3 | 3 | 8.2±4 | 0.996 |
| Y | Enrofloxacin | 101±3 | 3 | 3.6±3 | 0.995 |
| Y | Hydrocortisone | 26±10 | 9 | 5.4±10 | 0.988 |
| Y | Norfloxacin | 113±4 | 4 | -2.6±4 | 0.993 |
| Y | Prednisolone | 95±4 | 4 | 0.73±4 | 0.991 |
| Y | Sarafloxacin | 68±8 | 8 | -3.9±8 | 0.994 |
| Z | Amoxicillin | 58±4 | 4 | 5.8±4 | 0.995 |
| Z | Penicillin G | 43±13 | 13 | -0.003±13 | 0.991 |
| Z | Chlortetracycline | 42±82 | 70 | -42±41 | 0.965 |
| Z | Doxycycline | 71±18 | 18 | 19±21 | 0.976 |
| Z | Methacycline | 180±28 | 28 | 22±35 | 0.952 |
| Z | Oxytetracycline | 153±15 | 15 | 0.23±15 | 0.991 |
| Z | Tetracycline* | 105±12 | 12 | 10±13 | 0.994 |

For this method, the individual isotope labelled internal standards were chosen based on structural similarities (Table 5). Whenever possible an identical isotope labelled standard was chosen for target quantification. All the corticosteroids were tested both with prednisolone-D_8_ and cortisol-D_4_. For hydrocortisone, cortisol-D_4_ was chosen as internal standard. Both trimethoprim and sulfamethoxazole were analysed with sulfadiazine-^13^C_6_ as internal standard.

**S6. Matrix effect of the validated analytes**

For the sixteen accepted compounds, matrix effects (ME%) were tested by spiking known concentrations of target compounds in uncontaminated biogas digestate. Five replicates of both blank matrix (i.e., extracted digestate with no added STDs or ISTDs) and 20% MeOH (solvent) were prepared. Each of the samples were spiked with STDs corresponding to level 3 in the matrix matched calibration curve to make five replicates of matrix matched samples (MM_S_, equation 4) and solvent matched samples (MS_S_), respectively. In addition, non-spiked samples of both blank matrix and solvent were prepared (MM_0_ and MS_0_, n = 1). After analysis, the arithmetic means of the measurements were inserted in equation 4 to calculate the matrix effect.

$ME \left( \% \right)=\left[ \frac{\left( \overline{{MM}_{S}}-{MM}_{0} \right)}{\left( \overline{{MS}_{S}}-{MS}_{0} \right)}-1 \right]*100$ (eq. 5)

Efficiency of the extraction method (EEM) was calculated in the same manner as recovery (equation 5), except that the signal of the ISTDs were not included.

$EEM \left( \% \right) = \sum_{i=1}^{n=6} \frac{\left( {signal \left( matrix \right)}_{i}-signal\left( blank \right) \right)*conc\left( solvent \right)}{signal\left( solvent \right) * conc(matrix)}* \frac{100}{n}$ (eq. 6)

Table S9 shows the matrix effect of the validated compounds. When the matrix effect is positive, it indicates that the compound experiences a signal enhancement due to the digestate matrix, while a negative value indicates ion suppression. Some small amount of matrix effect is expected, and within a range of ±20% it is acceptable to omit matrix effect compensating measures. As can be seen from Table S9, the matrix effect was considerable for most of the validated compounds. Therefore, a matrix matched calibration curve was applied for the quantification of the individual target substance to compensate for matrix associated responses on the quantification signal in the LC/MS-MS quantification method.

The efficiency of the extraction method (EEM) was calculated by comparing the signal in spiked matrix samples (spiked before sample work up) with the signal in spiked solvent (no sample work up). The EEM expresses the combined effect of the matrix effect and the extraction efficiency. For the validated compounds, EEM varies from 9 to 192%. This demonstrates some of the difficulty in validating multi-compound methods and highlight the need to include a matrix matched calibration curve where the analytes are added before sample preparation.

**S7. Chromatography**


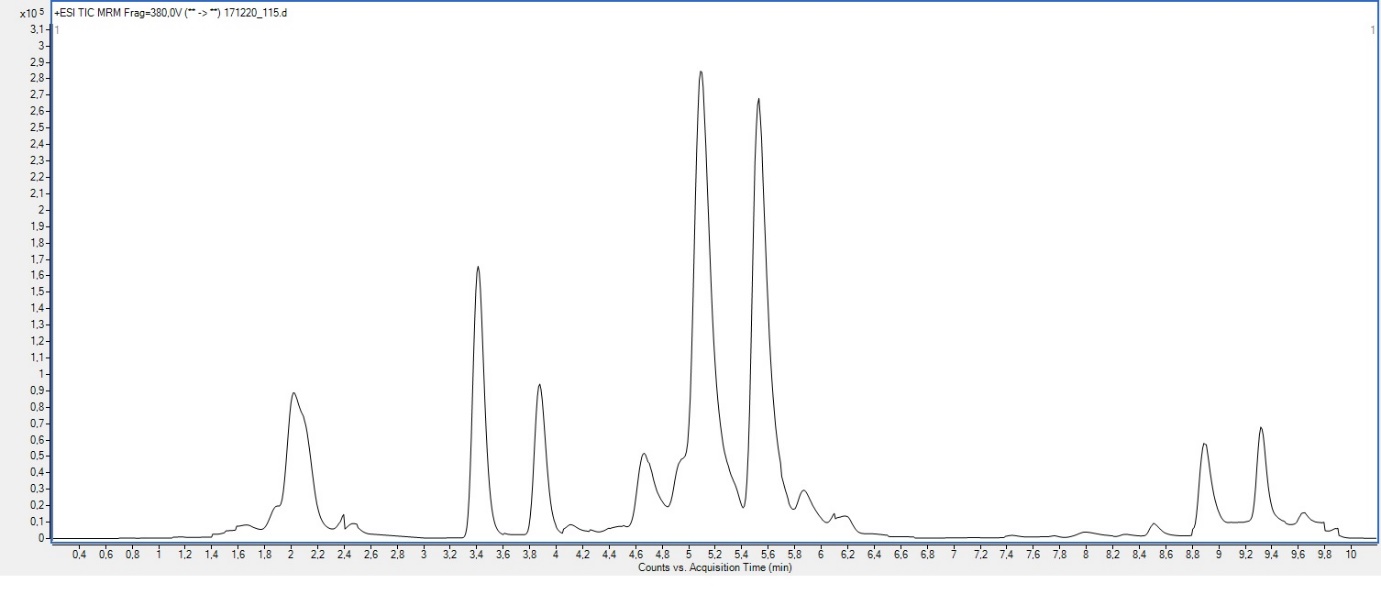


**Figure S1.** Total ion chromatogram of 15 ng mL^-1^ for X-compounds, 150 ng mL^-1^ for Y-compounds and 300 ng mL^-1^ for Z-compounds

*
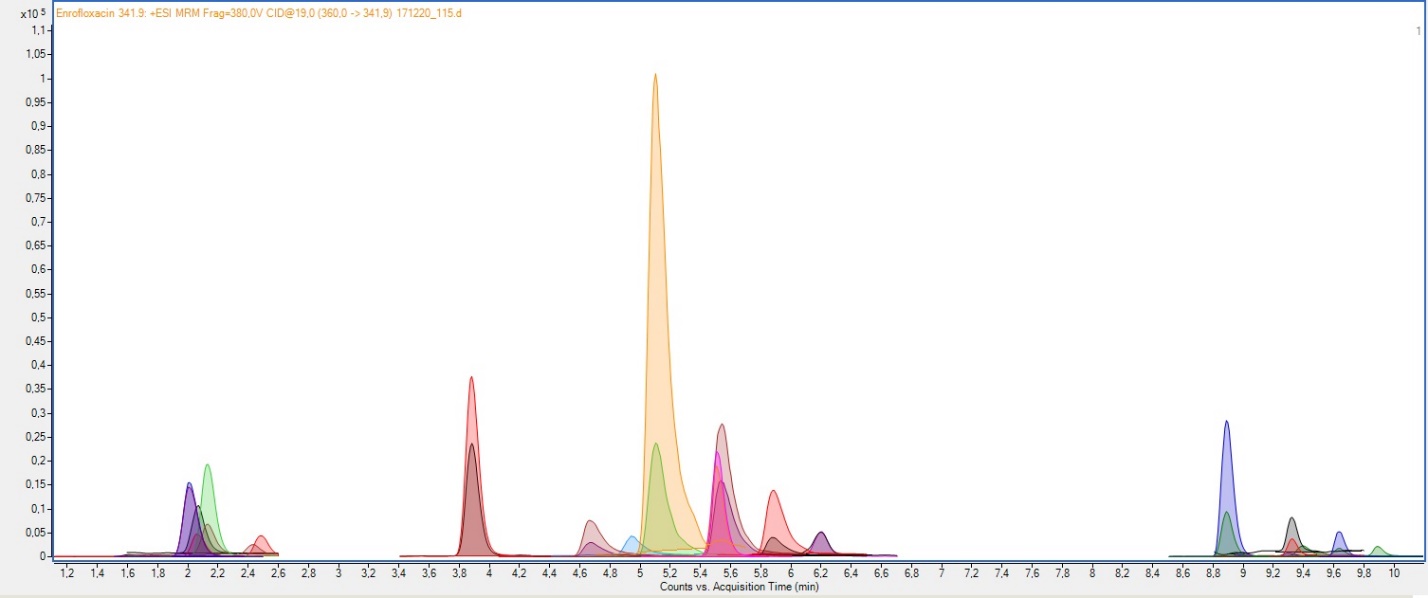
*

**Figure S2.** MRM-transitions for all successfully validated compounds at 15 ng mL^-1^ for X-compounds, 150 ng mL^-1^ for Y-compounds and 300 ng mL^-1^ for Z-compounds. The quantitative and qualitative MRM transition chosen in the validation is shown.


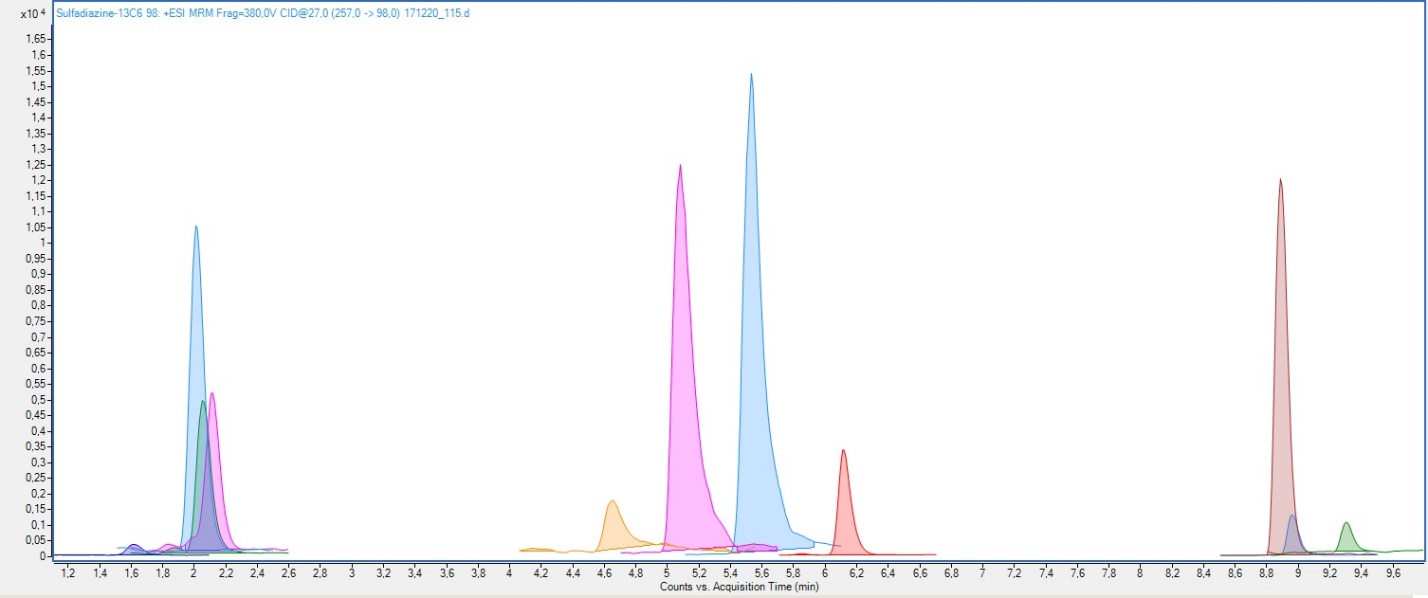


**Figure S3.** MRM-transitions for all successfully validated internal standards at 7.5 ng mL^-1^ for X-compounds, 75 ng mL^-1^ for Y-compounds and 150 ng mL^-1^ for Z-compounds. The transition chosen to calculate concentrations for each internal standard is shown.

**S8 Operating conditions at the biogas plants**

**Table S7.** Operating conditions at the 14 biogas plants. Reactor T: Temperature in the biogas reactor. Retention time: Retention time in the biogas reactor. THP: Thermal hydrolysis, i.e. the slurry is treated at 160°C at 3.5 bar prior to anaerobic digestion. The labelling of the biogas plants follow that of Ali et al. (2019), but are here ordered after substrate.

| **Plant** | **Solid /  Liquid** | **Substrate** | **Reactor  T (°C)** | **Retention time (days)** | **Thermal Pre treatment** | **% dry matter** |
| --- | --- | --- | --- | --- | --- | --- |
| E | L + S | Food waste | 39-41 | 35 | THP | 3.0 / 26.6 |
| G | L | Food waste | 39-40 | 20 | THP | 3.5 |
| K | L + S | Food waste | 52-53 | 15-20 | No | 2.5 / 34.8 |
| D | S | Sewage sludge | 37 | 20 | No | 47.4 |
| H | S | Sewage sludge | 54 | 12-15 | 70°C | 26.1 |
| J | S | Sewage sludge | 40 | 20-25 | THP | 33.1 |
| I | S | Sewage sludge | 55 | 14 | No | 31.6 |
| F | L + S | 45% food waste, 55% sewage sludge | 62 | 20 | 70°C | 1.6 / 21.2 |
| A | L + S | 45% food waste, 53% sewage sludge and 2% fish silage | 40 | 12-16 | THP | 1 / 27 |
| B | S | 85% sewage sludge, 15% food waste | 38 | 40 | THP | 26.2 |
| L | S + L | 60% sewage sludge, 12% septic, 28% food waste and fats | 40 | 20 | THP | 4.8 / 38.9 |
| C | L | 27% manure (swine and cattle), 72% food waste | 38-40 | 30-35 | 70°C | 4.8 |
| M | L | Slurry from organic milk cows | 35 | 30 | No | 4.3 |
| I_exp_ | Substrate and digestate | 20% sludge from young fish, 80% manure | 40 | 20 | No |  |

| **Substance** | **Endpoint** | **Days /hours** |  | **Test organism** | **Concentration  (μg kg^-1^ dw)** | **AF** | **PNEC  (μg kg^-1^ dw)** | **Reference** |
| --- | --- | --- | --- | --- | --- | --- | --- | --- |
| Amoxicillin | Metabolic activity | 8 h | EC50 | *Bacillus amyloliquefaciens* | 466 | 1000 | 0.466 | Menz et al. (2018) |
|  | Metabolic activity | 8 h | EC50 | *Pseudomonas putida* | 61 500 |  |  | Menz et al. (2018) |
| Penicillin G | Metabolic activity | 8 h | EC50 | *Bacillus amyloliquefaciens* | 171 | 1000 | 0.171 | Menz et al. (2018) |
|  | Metabolic activity | 8 h | EC50 | *Pseudomonas putida* | 279 000 |  |  | Menz et al. (2018) |
| Sulfadiazine | Metabolic activity | 8 h | EC50 | *Bacillus amyloliquefaciens* | >1 000 000 |  |  | Menz et al. (2018) |
|  | Metabolic activity | 8 h | EC50 | *Pseudomonas putida* | 5 610 | 1000 | 5.61 | Menz et al. (2018) |
|  | Iron reduction | 5 d | ED50 | Soil microorganisms | 47 553 |  |  | Thiele-Bruhn (2005) |
|  | Root elongation | 2 d | IC50 | *Wheat* | 28 100 |  |  | Jin et al. (2009) |
|  | Root elongation | 3 d | IC50 | *Chinese cabbage* | 31 300 |  |  | Jin et al. (2009) |
|  | Root elongation | 5 d | IC50 | *Tomato* | 92 900 |  |  | Jin et al. (2009) |
| Sulfamethazine | Iron reduction | 5 d | ED50 | Soil microorganisms | 75 149 |  |  | Thiele-Bruhn (2005) |
|  | Soil respiration | 2 d | EC10 | Soil microorganisms | 13 000 |  |  | Liu et al. (2009) |
|  | Root length  Root length | 20 d  20 d | NOAEL NOAEL | Rice Cucumber | 1 000  100 000 | 100 | 10 | Liu et al. (2009) Liu et al. (2009) |
|  | Seedling height Seedling height | 20 d  20 d | NOAEL NOAEL | Rice  Cucumber | 70 000  100 000 |  |  | Liu et al. (2009) Liu et al. (2009) |
| Ciprofloxacin | Nitrification | 28 d | NOAEL | Soil microorganisms | 100 000 |  |  | Parente et al. (2018) |
|  | Cumulative respiration | 28 d | NOAEL | Soil microorganisms | 1 000 000 |  |  | Parente et al. (2018) |
|  | Leaf growth | 9-16 d | NOAEL | *Lolium perenne* (ryegrass) | 10 000 000 |  |  | Parente et al. (2018) |
|  | Root growth | 9-16 d | NOAEL | *Lolium perenne* (ryegrass) | 10 000 000 |  |  | Parente et al. (2018) |
|  | Stem growth | 9-16 d | NOAEL | *Raphanus sativus* (radish) | 1 000 000 |  |  | Parente et al. (2018) |
|  | Root growth | 9-16 d | NOAEL | *Raphanus sativus* (radish) | 1 000 000 |  |  | Parente et al. (2018) |
|  | Leaf growth | 9-16 d | NOAEL | *Allium cepa* (onion) | 5 000 000 |  |  | Parente et al. (2018) |
|  | Root growth | 9-16 d | NOAEL | *Allium cepa* (onion) | 1 000 000 |  |  | Parente et al. (2018) |
|  | Micronucleus induction | 3 d | NOAEL | *Vicia faba* | 50 | 100 | 0.5 | Khadra et al. (2012) |

**S9 Ecotoxicity tests and calculation of predicted no effect concentration (PNEC) values

Table S8.** Inhibition of soil organisms by antibiotics. NOAEL = No observed adverse effect level. EC50 = Effect concentration 50, i.e. the measured endpoint is reduced by 50% compared to the control. IC50 = Inhibiting concentration 50. ED50 = Effective dose 50. No soil toxicity studies were found for ciprofloxacin and prednisolone. Aquatic studies for these compounds can be found in table S4.

**Table S9.** Inhibition of aquatic organisms by prednisolone. LOEC = Lowest observed effect concentration. EC50 = Effective concentration 50, i.e. the measured endpoint is reduced by 50%. LC50 = Lethal concentration 50, i.e. 50% of the population dies.

| **Endpoint** | **Days** |  | **Test organism** | **Concentration  (μg l^-1^)** | **AF** | **PNEC (μg l^-1^)** | **Reference** |
| --- | --- | --- | --- | --- | --- | --- | --- |
| Mortality |  | LC50 | *Brachionus calcyciflorus* | 22 290 |  |  | DellaGreca et al. (2004) |
| Mortality |  | LC50 | *Thamnocephalus platyrus* | 23% at 140 000 |  |  | DellaGreca et al. (2004) |
| Immobilization |  | EC50 | *Daphnia magna* | >85 000 |  |  | DellaGreca et al. (2004) |
| Cell density |  | EC50 | *Pseudokirchneriella subcapita* | >160 000 |  |  | DellaGreca et al. (2004) |
| Growth inhibition |  | EC50 | *Ceriodaphnia duba* | 230 |  |  | DellaGreca et al. (2004) |
| Developmental abnormalities | 118, F2 generation. | EC10 ≈ NOEC | *Physa acuta* | 1.6 | 100 | 0.016 | Bal et al. (2017) |

**Calculation of Predicted No Effect Concentration (PNEC)**

The calculation of PNEC follows the procedure from the Technical Guidance Document on Risk Assessment from the European Chemicals Bureau (2003), the procedure is described in the following:

1. Collect terrestrial ecotoxicity data and convert into µg kg^-1^ dry weight soil.

2. In cases where there are no terrestrial ecotoxicity data, or there are data on only one organism, aquatic ecotoxicity data are collected as well. This is the case for prednisolone (no data).

3. PNEC_soil_ can be calculated in two different ways:

**A. PNEC calculation by the use of assessment factors.**This method applies to compounds when toxicity data are available for a producer, a consumer and/or a decomposer in soil.

$\frac{EC50 or NOAEL or LC50 \left[ \mu g {kg}^{-1} \right]}{AF}={PNEC}_{SOIL}\left[ \mu g {kg}^{-1} \right]$ (eq. 7)

Where AF is the assessment factor appropriate for the toxicity data. The AF is meant to reflect the uncertainty with predicting ecosystem effects from laboratory tests on a limited number of species, often for a limited duration.
 **Table S10.** Overview of the assessment factors. The table is adapted from table 20 in the Technical Guidance Document on Risk Assessment of the European Commission (2003).

| **Information available** | **Assessment factor** |
| --- | --- |
| LC50 / EC50 short term toxicity test(s) | 1000 |
| NOAEL for one long-term toxicity test | 100 |
| NOAEL for additional long-term toxicity tests of two trophic levels | 50 |
| NOAEL for additional long-term toxicity tests for three species of three trophic levels | 10 |

*Example of calculation*: For sulfadiazine, there are only short-term acute toxicity tests. Therefore, an assessment factor of 1000 is used: PNEC_soil_  = 5610 μg kg^-1^ dw / 1000 = 5.61 μg kg^-1^.

**B. PNEC calculation by the use of the equilibrium partitioning method**
When there are no terrestrial toxicity data, or only data from one species, the equilibrium partitioning method can be used. PNEC_WATER_ is calculated as in section A, with the unit μg l^-1^. Thereafter, the PNEC_WATER_ is converted into PNEC_SOIL_ by equation 1 (adapted from equation 72 in European Commission (2003)).

${PNEC}_{soil}\left[ \mu g {kg}^{-1} \right]=\frac{K_{d}[-]}{{RHO}_{soil \left[ kg * l^{-1} \right]}}*{PNEC}_{water}\left[ \mu g l^{-1} \right]$ (eq. 8)

Where K_d_ is the partition coefficient soil water, and RHO_SOIL_ is the bulk density of soil. K_d_ is calculated by equation 2, as recommended by the European Commission (2003).

$K_{d}\boldsymbol{=}K_{OC}* f_{OC}*10$ (eq. 9)

For prednisolone, K_OC_ is modelled to 36.36 by EPISUITE. The European Commission (2003) use 0.02 as a standard value for f_OC_. K_d_ for prednisolone is then:

$$K_{d}=36.36*0.02*10=7.272$$

The calculation of K_d_ from K_OC_ is primarily meant to be used on neutral species, as the main sorption mechanism for these in soil is partitioning into organic matter. Prednisolone can ionise, and therefore the K_d_ is multiplied with 10 to account for electrostatic sorption as recommended by European Commission (2003). Further, PNEC_SOIL_ is calculated by equation 1. RHO_SOIL_ is set to 1.3 kg l^-1^, as a typical bulk density of soil.

$${PNEC}_{SOIL}= \frac{0.016 \mu g l^{-1}*7.272}{1.3 kg l^{-1}}=0.224 \mu g {kg}^{-1}dw$$

Note that the calculation of PNEC_SOIL_  for prednisolone has significant uncertainties, due to lack of soil ecotoxicity data and measurements of K_d_.

**Calculation of PNEC values for selection antibiotic resistant bacteria**

Menz et al. (2019) calculated PNEC values for selection of antibiotic resistant bacteria from *in vitro* antimicrobial susceptibility data (MICs). For the detailed calculation procedure, please read Menz et al. (2019). These PNEC values are given as μg l^-1^ soil pore water. To compare with the PEC_SOIL_ values calculated in the present study, the PNEC values must be converted into μg kg^-1^ dry soil. The equilibrium partitioning method suggested for aquatic ecotoxicity data (see section 3B under *Calculation of PNEC*) are used, with the addition of a term for the fraction of pore water in soil (equation 3):

${PNEC}_{soil}\left[ \mu g {kg}^{-1} \right]=\frac{K_{d}\left[ - \right]}{{RHO}_{soil \left[ kg * l^{-1} \right]}}*{PNEC}_{pore water}\left[ \mu g l^{-1} \right]*f_{pw}$ (eq. 10)

Where pw = pore water. As in other parts of the current study, RHO_SOIL_ is set to 1.3 kg l^-1^. The fraction of pore water, f_PW_ is set to 0.25 (v/v). For the calculation of PNEC, the Kd, and K_OC_ values given in Table S5 were used. For amoxicillin and penicillin G, K_d_ are estimated from K_OC_ by equation 2. For sulfadiazine and ciprofloxacin, K_d_ is estimated as the average of the minimum and maximum K_d_ values reported in Table S5. Further, PNEC_SOIL_ is estimated by equation 3 as in table S6.
 **Table S11.** Log K_OW_, K_d_ and K_OC_ as reported by Cycoń et al. (2019)

|  | Kd (l kg^-1^) | K_OC_ (l kg^-1^) |
| --- | --- | --- |
| Amoxicillin | - | 865.5 |
| Penicillin G | - | 2.68 |
| Sulfadiazine | 1.40-14 | 37-125 |
| Ciprofloxacin | 427-4,844 | 1,127-61,000 |

**Table S12.** Estimation of PNEC_SOIL_ for selection of antibiotic resistant bacteria from experimental data provided by Cycoń et al. (2019).

|  | PNEC_PW_, Menz et al. (2019) | K_OC_ | Kd | PNEC_SOIL_ |
| --- | --- | --- | --- | --- |
| Amoxicillin | 0.20-0.25 | 865.5 | 865.5*0.02*10 = 173.1 | (0.20-0.25)*173.1*0.25/1.3 = 6.7-8.3 |
| Penicillin G | 0.25 | 2.68 | 2.68*0.02*10 = 0.536 | 0.25*0.536*0.25/1.3 = 0.026 |
| Sulfadiazine | 21 |  | (1.4+14)/2 = 7.7 | 21*7.7*0.25/1.3 = 31 |
| Ciprofloxacin | 0.064 |  | (427+4,844)/2 = 2624.5 | 0.064*2624.5*0.25/1.3 = 32 |

**S10. Description of the performed statistics**

**Correlation analysis**
Pearson correlation analysis were used to explore the numerical part of the data set, i.e. the levels of pharmaceuticals (Table 1) and the following operating conditions (Table S1): Substrate (fraction of food waste, sewage sludge, manure, fish sludge), Retention time, Reactor temperature, Dry matter of the digestate. The qualitative operating conditions pre-treatment, polymer addition and precipitant addition were left for analysis of variance. Values below LOQ was replaced with ½ LOQ, while values below the detection limit (LOD) was replaced with ½ LOD.

**Figure S4.** Pearson correlation analysis. Blue circles means there is a positive correlation, red circles means there is a negative correlation. Only significant correlations (p < 0.05) are shown. The size of the circle and the darkness of the colour increases with the correlation coefficient (colour legend to the right). Total: sum of pharmaceuticals in each digestate.


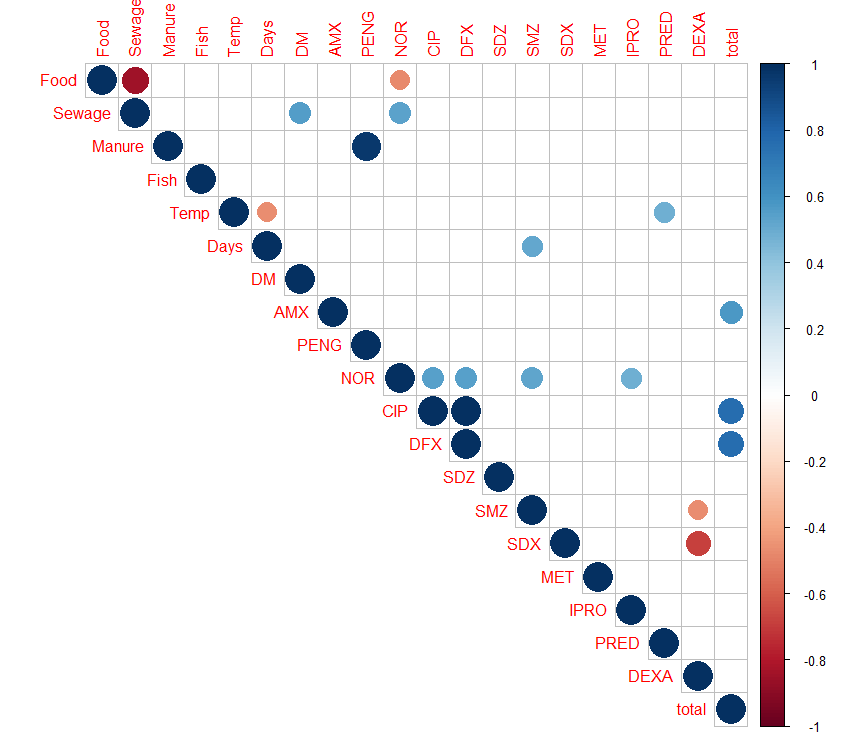


There is a positive correlation between the fluoroquinolones norfloxacin, ciprofloxacin, and difloxacin. For the other pharmaceuticals there are no correlations within the groups. Note that the correlations are strongly affected by individual observations. For example, the Penicillin G concentration is strongly positively correlated with the manure content of the substrate. This can be explained as the only digestate where Penicillin G was present was the only digestate using 100% manure as substrate.

**References**

Ali, A.M., Nesse, A.S., Eich-Greatorex, S., Sogn, T.A., Aanrud, S.G., Aasen Bunæs, J.A., Lyche, J.L., Kallenborn, R., 2019. Organic contaminants of emerging concern in Norwegian digestates from biogas production. Environ. Sci. Process. Impacts 21, 1498–1508.

Bal, N., Kumar, A., Nugegoda, D., 2017. Assessing multigenerational effects of prednisolone to the freshwater snail, Physa acuta (Gastropoda: Physidae). J. Hazard. Mater. 339, 281–291.

Cycoń, M., Mrozik, A., Piotrowska-seget, Z., 2019. Antibiotics in the Soil Environment — Degradation and Their Impact on Microbial Activity and Diversity. Front. Microbiol. 10, 1–45.

DellaGreca, M., Fiorentino, A., Isidori, M., Lavorgna, M., Previtera, L., Rubino, M., Temussi, F., 2004. Toxicity of prednisolone, dexamethasone and their photochemical derivatives on aquatic organisms. Chemosphere 54, 629–637.

European Commission, 2003. Technical Guidance Document on Risk Assessment in Support of Commission Directive 93/67/EEC, Commission Regulation (EC) No 1488/94, and of Directive 98/8/EC. Part II. Luxembourg.

Hu, X.Y., Zhou, Q., Luo, Y., 2010. Occurence and source analysis of typical veterinary antibiotics in manure, soil, vegetables and groundwater from organic vegetable bases, northern China. Environ. Pollut. 158, 2992–2998.

Jin, C., Chen, Q., Sun, R., Zhou, Q., Liu, J., 2009. Eco-toxic effects of sulfadiazine sodium, sulfamonomethoxine sodium and enrofloxacin on wheat, Chinese cabbage and tomato. Ecotoxicology 18, 878–885.

Khadra, A., Pinelli, E., Lacroix, M.Z., Bousquet-Melou, A., Hamdi, H., Merlina, G., Guiresse, M., Hafidi, M., 2012. Assessment of the genotoxicity of quinolone and fluoroquinolones contaminated soil with the Vicia faba micronucleus test. Ecotoxicol. Environ. Saf. 76, 187–192.

Liu, F., Ying, G.G., Tao, R., Zhao, J.L., Yang, J.F., Zhao, L.F., 2009. Effects of six selected antibiotics on plant growth and soil microbial and enzymatic activities. Environ. Pollut. 157, 1636–1642.

Menz, J., Müller, J., Olsson, O., Kümmerer, K., 2018. Bioavailability of Antibiotics at Soil-Water Interfaces: A Comparison of Measured Activities and Equilibrium Partitioning Estimates. Environ. Sci. Technol. 52, 6555–6564.

Menz, J., Olsson, O., Kümmerer, K., 2019. Antibiotic residues in livestock manure: Does the EU risk assessment sufficiently protect against microbial toxicity and selection of resistant bacteria in the environment? J. Hazard. Mater.

Parente, C.E.T., Sierra, J., Martí, E., 2018. Ecotoxicity and biodegradability of oxytetracycline and ciprofloxacin on terrestrial and aquatic media. Orbital 10, 262–271.

Thiele-Bruhn, S., 2005. Microbial inhibition by pharmaceutical antibiotics in different soils - dose-response relations determined with the iron (III) reduction test. Environ. Toxicol. Chem. 24, 869–876.
